# Single-cell and functional profiling identifies an IL6-centered immunometabolic communication circuit in multiple myeloma

**DOI:** 10.3389/fimmu.2026.1734500

**Published:** 2026-07-27

**Authors:** Delong Lang, Jing Wu, Jiayou Zhang, Yuhu Feng

**Affiliations:** 1 Department of Hematology, Fuyang People’s Hospital (The Affiliated Fuyang People’s Hospital of Anhui Medical University), Fuyang, Anhui, China; 2 Department of Hematology, The Second Affiliated Hospital of Chengdu Medical College, China National Nuclear Corporation 416 Hospital, Chengdu, Sichuan, China

**Keywords:** bone marrow microenvironment, CellChat, IL-6, immunometabolism, JAK–STAT3, metabolic vulnerability, multiple myeloma, tumor-associated macrophages

## Abstract

Multiple myeloma (MM) persists within a specialized bone marrow niche in which malignant plasma cells, immune dysfunction, inflammatory signaling, and metabolic stress reinforce one another. To resolve this ecosystem at compartment-level resolution, we integrated public single-cell RNA sequencing data with pathway scoring, cell-cell communication inference, independent clinical validation, multiplex immunofluorescence, and metabolic perturbation experiments. Analysis of 95,940 bone marrow cells identified 32 annotated populations and revealed broad microenvironmental remodeling in MM, including plasma-cell expansion, altered cytotoxic and dendritic-cell compartments, TAM-associated inflammatory programs, and lineage-specific hematopoietic perturbations. Hallmark pathway analysis identified recurrent immunometabolic programs, including IL6-JAK-STAT3, TNFα-NFκB, mTORC1 signaling, oxidative phosphorylation, unfolded protein response, hypoxia, and checkpoint/exhaustion-associated pathways. CellChat analysis showed disease-associated rewiring of ligand-receptor networks involving malignant plasma cells, TAMs, dendritic cells, and T/NK subsets, with checkpoint-enriched communication and clinically relevant plasma-cell and CD274 survival associations. Serum IL-6 was elevated in an independent clinical validation cohort. Multiplex immunofluorescence confirmed PD-L1-positive plasma cells, C1QA/C1QB/C1QC-positive TAMs expressing LAG3, and CD8-positive/LAG3-positive cytotoxic T cells. Mechanistically, malignant plasma cells showed transcriptional activation of LDH-associated glycolytic/lactate programs and ASCT2/SLC1A5-GLS-linked glutamine-metabolic programs, nominating these pathways as functional vulnerabilities. Accordingly, the LDH inhibitor galloflavin and the ASCT2/SLC1A5 glutamine-transport inhibitor V-9302 suppressed RPMI-8226 viability in dose- and time-dependent manners; their combination produced synergistic anti-myeloma activity supported by Bliss synergy, combination-index analysis, observed-versus-expected inhibition, and apoptosis-related validation. Together, these findings support an IL6-centered immunometabolic communication circuit linking malignant plasma cells, TAMs, and dysfunctional T cells, and identify cooperative glycolytic/lactate and glutamine-dependent metabolic vulnerabilities with therapeutic relevance in MM.

## Introduction

Multiple myeloma (MM) remains a clinically challenging plasma-cell malignancy in which disease persistence is shaped not only by malignant plasma cells but also by the bone marrow microenvironment. Increasing evidence indicates that metabolic stress, immune dysfunction, and altered intercellular communication cooperate to generate a tumor-permissive niche in MM (1–4). Immune-checkpoint engagement and T-cell dysfunction further contribute to immune escape and treatment resistance (5–7). However, how inflammatory, metabolic, and checkpoint-associated programs are coordinated across individual bone marrow compartments remains incompletely defined.

IL6-JAK-STAT3 signaling represents a central inflammatory axis linking tumor-cell survival, myeloid activation, and immune regulation in cancer and MM (8–10). This pathway is particularly relevant in MM because malignant plasma cells, macrophage-lineage cells, and exhausted lymphocytes coexist within a cytokine-rich and metabolically constrained marrow niche. Previous single-cell and communication-based studies have begun to characterize immune suppression and ligand-receptor remodeling in MM (11, 12), yet most analyses have considered cellular composition, pathway activity, and intercellular communication as separate layers. A compartment-resolved framework connecting these layers to clinical and functional validation is still needed.

Here, we constructed an integrated single-cell transcriptomic atlas of the MM bone marrow ecosystem and analyzed 95,940 cells from MM and healthy bone marrow samples. We combined lineage-resolved clustering, sample-level cell proportion analysis, Hallmark pathway scoring, and CellChat-based ligand-receptor inference with independent clinical IL-6 validation, survival analyses, multiplex immunofluorescence, and metabolic perturbation experiments. This strategy was designed to determine whether MM is sustained by a coordinated immunometabolic circuit linking malignant plasma cells, tumor-associated macrophages (TAMs), and dysfunctional T cells.

Our analysis identifies broad cellular and communication remodeling in MM, including plasma-cell expansion, TAM-associated inflammatory programs, subset-specific T/NK-cell dysfunction, checkpoint-enriched communication, and recurrent activation of IL6-JAK-STAT3-associated immunometabolic pathways. Malignant plasma cells also exhibited transcriptional activation of LDH-associated glycolytic/lactate programs and ASCT2/SLC1A5-GLS-linked glutamine-metabolic programs, providing a mechanistic rationale for pharmacologic testing of the LDH inhibitor galloflavin and the ASCT2/SLC1A5 glutamine-transport inhibitor V-9302 (13–16). Clinically, plasma-cell-associated features, CD274 expression, and elevated serum IL-6 supported the relevance of this inflammatory-checkpoint axis. Functionally, concurrent targeting of glycolytic/lactate and glutamine-dependent metabolic pathways produced synergistic anti-myeloma activity, consistent with cooperative metabolic dependencies inferred from the single-cell data (13–18). Together, these findings support an IL6-centered immunometabolic circuit in which malignant plasma cells, TAMs, and dysfunctional T cells reinforce immune suppression and metabolic vulnerability in MM.

## Methods

### Study design and data sources

This study was designed to define immunometabolic remodeling and cell–cell communication changes in the MM bone marrow microenvironment by integrating public single-cell transcriptomic data with independent clinical validation, spatial immunophenotyping, and *in vitro* metabolic perturbation experiments. The primary single-cell analysis focused on healthy volunteer/control (HV) and MM bone marrow samples. Serum interleukin-6 (IL-6), multiplex immunofluorescence (mIF), drug-response, and apoptosis-related experiments were used as independent validation layers rather than as matched measurements from the same individuals included in the public scRNA-seq cohort.

### Single-cell RNA-seq data acquisition and preprocessing

Single-cell RNA-sequencing (scRNA-seq) count matrices from bone marrow samples were obtained from the Gene Expression Omnibus (GEO; accession GSE232988). Analyses were performed in R v4.3.0 using Seurat v5.0.1 unless otherwise specified. For the primary HV-versus-MM comparison, samples annotated as healthy volunteer/control or MM were retained. Cells with fewer than 200 detected genes or more than 20% mitochondrial unique molecular identifiers (UMIs) were excluded. Genes detected in fewer than three cells were removed. Raw counts were log-normalized, and the top 3,000 highly variable genes were identified using the variance-stabilizing transformation method.

To mitigate donor- and batch-associated variation, datasets were integrated using canonical correlation analysis (CCA)-based integration implemented in Seurat. Principal component analysis (PCA) was performed on the integrated expression matrix. The number of principal components used for downstream analyses was selected according to variance-explained and elbow-plot inspection. Uniform manifold approximation and projection (UMAP) was used for two-dimensional visualization. Graph-based clustering was performed using the Louvain algorithm at a resolution of 0.6 for the global atlas, followed by manual inspection of lineage marker genes.

### Cell-type annotation and global cell-composition analysis

Clusters were annotated using canonical immune, plasma-cell, myeloid, dendritic-cell, erythroid, platelet, and hematopoietic progenitor marker genes. Annotated compartments included T/NK cells, B cells, plasma cells, monocytes/macrophages, dendritic cells, hematopoietic stem/progenitor cells (HSPCs), erythroid-lineage populations, immature neutrophils, platelets, and minor or ambiguous populations. Marker genes for each cluster were identified using Seurat FindAllMarkers with the Wilcoxon rank-sum test, requiring min.pct = 0.25 and log2 fold-change >0.25, followed by multiple-testing correction using the Benjamini–Hochberg method unless otherwise stated.

Cell-type proportions were calculated at both cohort and sample levels. Cohort-level proportions were used for descriptive visualization of the global cellular landscape. For statistical comparison of cell-subset abundance between HV and MM, proportions were calculated per individual sample to avoid treating single cells as independent biological replicates. Sample-level group comparisons were performed using two-sided Wilcoxon rank-sum tests. Where multiple cell subsets were compared within the same analysis family, Benjamini–Hochberg false-discovery rate (FDR) correction was applied or nominal p values were reported explicitly as exploratory, as indicated in the figure legends.

### Subtype-specific reclustering

Major cellular compartments were subset from the global Seurat object and reclustered to resolve lineage-specific transcriptional states. T/NK cells were reclustered into 14 subsets, including CD4 activated memory T cells, CD4 cytotoxic T cells, CD4 memory T cells, CD4 naive T cells, CD8 activated T cells, CD8 cytotoxic T cells, CD8 memory T cells, CD8 naive T cells, CD56 bright NK cells, gamma-delta cytotoxic T cells, MAIT cytotoxic T cells, NK cells, NKT cells, and Tregs. Monocyte/macrophage-lineage cells were reclustered into CD14 monocytes, macrophages, and TAMs. B/plasma-lineage cells were reclustered into memory B cells, naive B cells, plasma cells, and PrePro B cells. Dendritic cells were reclustered into CD1C DCs, CD14 DCs, CLEC9A DCs, and plasmacytoid DCs. HSPC/blood-lineage cells were reclustered into erythroblasts, erythrocytes, erythroid progenitors, HSPCs, immature neutrophils, and platelets. For each lineage, marker genes were identified and visualized to confirm subtype identity.

### Differential expression and selected gene-module profiling

For each annotated cell subset, differential gene expression between MM and HV was assessed using Seurat FindMarkers. Results were interpreted in the context of subtype identity and cohort distribution. In addition to genome-wide differential expression, selected metabolism-, checkpoint-, and exhaustion-related genes were curated for focused visualization. Metabolic genes included representative markers of glutamine metabolism, glucose metabolism, and lipid metabolism, such as GLS, SLC1A5, HK2, LDHA, ENO1, GAPDH, TPI1, PKM, ACLY, FASN, SCD, SREBF1, and related genes. Checkpoint and exhaustion-associated genes included LAG3, TIGIT, HAVCR2, TOX, ENTPD1, PDCD1, CTLA4, and CD274 when detected. Average expression was summarized by cell subtype and cohort and visualized as row-scaled heatmaps using ComplexHeatmap v2.16.0 or pheatmap. These heatmaps were interpreted as subset-level expression summaries rather than direct evidence of uniform pathway activation across an entire lineage.

### Hallmark pathway scoring and visualization strategy

MSigDB Hallmark gene sets were used to evaluate pathway-level programs across cell subsets. Pathway activity was estimated using GSVA or GSEA-based approaches depending on the analysis level. For subset-level heatmaps, average expression matrices were generated for each cell subtype–cohort combination and scored using GSVA. Pathway scores were scaled for visualization. For differential enrichment based on differentially expressed genes, clusterProfiler v4.10.0 was used, and pathways with adjusted p values below 0.05 were considered statistically enriched.

To ensure pathway-selection transparency, all MSigDB Hallmark gene sets were initially evaluated. Pathways displayed in the main figures were selected according to predefined criteria: differential activity between MM and HV in at least one major cell compartment; magnitude of the MM–HV effect size; recurrence across related cell compartments; and biological relevance to the central hypothesis of MM immunometabolic remodeling. Displayed pathways therefore emphasize immune-inflammatory programs, including interferon response, TNFα–NFκB signaling, IL6–JAK–STAT3 signaling, inflammatory response, and complement; metabolic and stress-response programs, including oxidative phosphorylation, mTORC1 signaling, MYC targets, hypoxia, angiogenesis, protein secretion, and unfolded protein response; and marrow lineage-associated programs, including heme metabolism and coagulation. Full Hallmark pathway heatmaps for individual lineages are provided in the supplementary figures, whereas the integrated pathway dot plot summarizes recurrent pathway remodeling across major marrow compartments.

### Cell–cell communication analysis

Cell–cell communication networks were inferred using CellChat v1.6.1 (11). Normalized Seurat objects were converted into CellChat objects, and the CellChat human ligand–receptor database was used to estimate interaction probabilities among annotated cell types. Analyses were performed separately for HV and MM cohorts using the same cell-type annotation framework. Low-abundance populations were retained when sufficient cells were available for robust communication inference.

Global communication patterns were visualized using chord diagrams, circle plots, network graphs, and interaction-strength heatmaps. Interaction strength was summarized between sender and receiver cell types. Communication events were further classified into cytokine, checkpoint, and other interaction classes. The “other” category included adhesion-, extracellular-matrix-, integrin-, and metabolism-associated ligand–receptor interactions not assigned to the cytokine or checkpoint groups. Network-level comparisons were used to identify disease-associated communication patterns and representative ligand–receptor axes, including TNFRSF17-associated plasma-cell signaling, XCL1/XCL2–XCR1 interactions, CD40LG–CD40-related communication, and checkpoint-associated interactions involving PD-L1- or LAG3-related programs.

### Clinical data collection and serum IL-6 validation

Clinical variables and serum IL-6 measurements were obtained retrospectively from an independent clinical validation cohort treated at the Department of Hematology, Fuyang People’s Hospital, China. Written informed consent was obtained where required, and all data were de-identified before analysis. Demographic information, disease subtype, and laboratory results were extracted from electronic medical records. Serum IL-6 values were retrieved from the hospital laboratory information system as part of routine clinical testing; no additional blood sampling or experimental IL-6 assays were performed specifically for this study.

The serum IL-6 cohort was independent of the public scRNA-seq dataset and was not a matched validation cohort from the same individuals used for single-cell analysis. IL-6 data were therefore interpreted as an independent clinical validation layer supporting the inflammatory relevance of the IL6–JAK–STAT3 axis identified by transcriptomic and cell–cell communication analyses. IL-6 concentrations between MM and normal controls were compared using the Mann–Whitney U test.

### Multiplex immunofluorescence validation

Formalin-fixed, paraffin-embedded bone marrow or tissue blocks were retrieved retrospectively from Fuyang People’s Hospital. Sections of 4 μm thickness were mounted on charged slides, baked at 60 °C for 1 h, deparaffinized in xylene, and rehydrated through graded ethanol to water. Heat-induced epitope retrieval was performed in Tris–EDTA buffer (pH 9.0) at 95–98 °C for 20 min. Endogenous peroxidase activity was quenched with 3% hydrogen peroxide, and nonspecific binding was blocked before antibody incubation.

Multiplex immunofluorescence staining was performed using a sequential tyramide signal amplification workflow with Opal fluorophores. Each staining cycle consisted of primary antibody incubation, HRP-conjugated secondary antibody incubation, tyramide deposition, and heat-mediated antibody stripping before the next marker. Nuclei were counterstained with DAPI. Single-plex controls, fluorescence-minus-one controls, isotype controls, and no-primary controls were used to assess spectral overlap and background staining.

Antibody panels were designed to validate the inferred plasma-cell, macrophage/TAM, and dysfunctional T-cell compartments. Plasma-cell-associated staining included PD-L1 with IGHG1, XBP1, and MZB1. TAM-associated staining included C1QA, C1QB, C1QC, LAG3, and DAPI. Cytotoxic T-cell staining included CD8, LAG3, and DAPI. Slides were scanned using a multispectral imaging system at 20× magnification with fixed exposure settings within each batch. Spectral unmixing and autofluorescence subtraction were performed using single-stained controls. Representative non-necrotic regions of interest were selected by a hematopathologist blinded to clinical annotation.

Cells were segmented using DAPI-based nuclear segmentation with membrane/cytoplasmic expansion. Plasma cells were defined by co-expression of IGHG1, XBP1, and MZB1. TAMs were defined by C1QA/C1QB/C1QC positivity. Cytotoxic T cells were defined by CD8 positivity. PD-L1 or LAG3 positivity was determined using fluorescence-minus-one-derived intensity thresholds. Co-expression required both compartment identity and marker positivity within the same segmented cell.

### Rationale for metabolic inhibitor selection

Galloflavin and V-9302 were selected to test metabolic vulnerabilities nominated by the single-cell plasma-cell transcriptomic program. In the B/plasma-cell compartment, MM plasma cells showed increased expression of LDH-associated glycolytic/lactate genes, including LDHA and related glycolytic enzymes, together with glutamine-metabolism and glutamine-transport genes, including GLS and SLC1A5. Galloflavin is a pharmacologic inhibitor of lactate dehydrogenase (LDH), targeting the pyruvate-to-lactate step of glycolytic/lactate metabolism. V-9302 blocks ASCT2/SLC1A5-mediated glutamine transport, thereby targeting glutamine uptake and glutamine-dependent metabolic dependency. This drug pair was therefore selected to functionally test whether transcriptionally inferred glycolytic/lactate and glutamine-transport programs constitute cooperative metabolic vulnerabilities in MM cells (14–16, 19, 20).

### Cell culture and metabolic perturbation assays

RPMI-8226 human MM cells were cultured in RPMI-1640 medium supplemented with 10% fetal bovine serum and 1% penicillin-streptomycin at 37 °C in a humidified incubator with 5% CO2. Cells were used within 20 passages and routinely confirmed to be free of mycoplasma contamination. Galloflavin and V-9302 were obtained from MedChemExpress. Galloflavin was used as an LDH inhibitor targeting glycolytic/lactate metabolism, whereas V-9302 was used as an ASCT2/SLC1A5 glutamine-transport inhibitor targeting glutamine uptake. DMSO was used as the vehicle for compound stocks unless otherwise indicated. The final DMSO concentration was maintained at 0.1% across treatment conditions.

Cell viability was measured using the Cell Counting Kit-8 assay. RPMI-8226 cells were seeded into flat-bottom 96-well plates at 8 × 10³ cells per well and allowed to equilibrate overnight. Cells were treated with serial concentrations of galloflavin or V-9302 for 48 h. Galloflavin was tested at 10, 20, 40, 80, and 120 μM, and V-9302 at 2, 5, 10, 20, and 40 μM unless otherwise indicated. After treatment, CCK-8 reagent was added, plates were incubated for 2 h at 37 °C, and absorbance was measured at 450 nm. Corrected absorbance was calculated by subtracting blank absorbance, and viability was normalized to DMSO-treated controls. Dose–response curves were fitted by nonlinear regression, and IC50 values were estimated from the fitted curves.

Drug-combination effects between galloflavin and V-9302 were evaluated using normalized inhibition values. The Bliss independence model was used to calculate expected inhibition under non-interaction assumptions and to derive Bliss synergy scores. Observed inhibition was compared with Bliss-expected inhibition across tested dose combinations. Approximate combination index values were calculated as an additional interaction metric; values below 1 were interpreted as synergistic, values near 1 as additive, and values above 1 as antagonistic. Combination effects were summarized using observed-inhibition matrices, Bliss-score heatmaps, combination-index visualizations, and observed-versus-expected inhibition plots.

For apoptosis-related validation treated and control cells were harvested for western blotting of Bax and Bcl-2, with GAPDH as loading control. Flow-cytometry apoptosis analysis was performed using an Annexin V/propidiumiodide or equivalent apoptosis-detection workflow according to the manufacturer’s protocol. Apoptotic fractions were quantified using standard gating strategies.

### Survival analysis

Survival analyses were performed using public MM cohorts. CD274-related survival analysis was performed using the MMRF–TCGA resource, whereas survival analyses for other molecular or immune-cell-associated features were performed using GSE136400. Patients with missing survival time or missing required covariates were excluded from the relevant analysis. For visualization, patients were stratified into high- and low-feature groups using the indicated cut-off strategy. Kaplan–Meier curves were generated using the survival and survminer packages, and groups were compared using log-rank tests. Where appropriate, Cox proportional-hazards models were used to estimate hazard ratios and 95% confidence intervals. Proportional-hazards assumptions were assessed using Schoenfeld residuals.

### Statistical analysis and data visualization

All statistical analyses were performed in R v4.3.0 unless otherwise specified. Data visualization was performed using ggplot2, ComplexHeatmap, pheatmap, circlize, Seurat, CellChat, and related R packages. Unless otherwise indicated, tests were two-sided, and p < 0.05 was considered statistically significant. For multi-feature analyses, p values were adjusted using the Benjamini–Hochberg method where applicable. For cell-proportion comparisons, statistical tests were performed at the sample level. For continuous clinical variables such as serum IL-6, nonparametric tests were used when distributional assumptions were not met. For *in vitro* assays, replicate structure and statistical tests are reported in the corresponding figure legends. Effect sizes, p values, and confidence intervals are reported where relevant.

## Results

### Single-cell landscape reveals global cellular remodeling in multiple myeloma bone marrow

**Figure 1:**
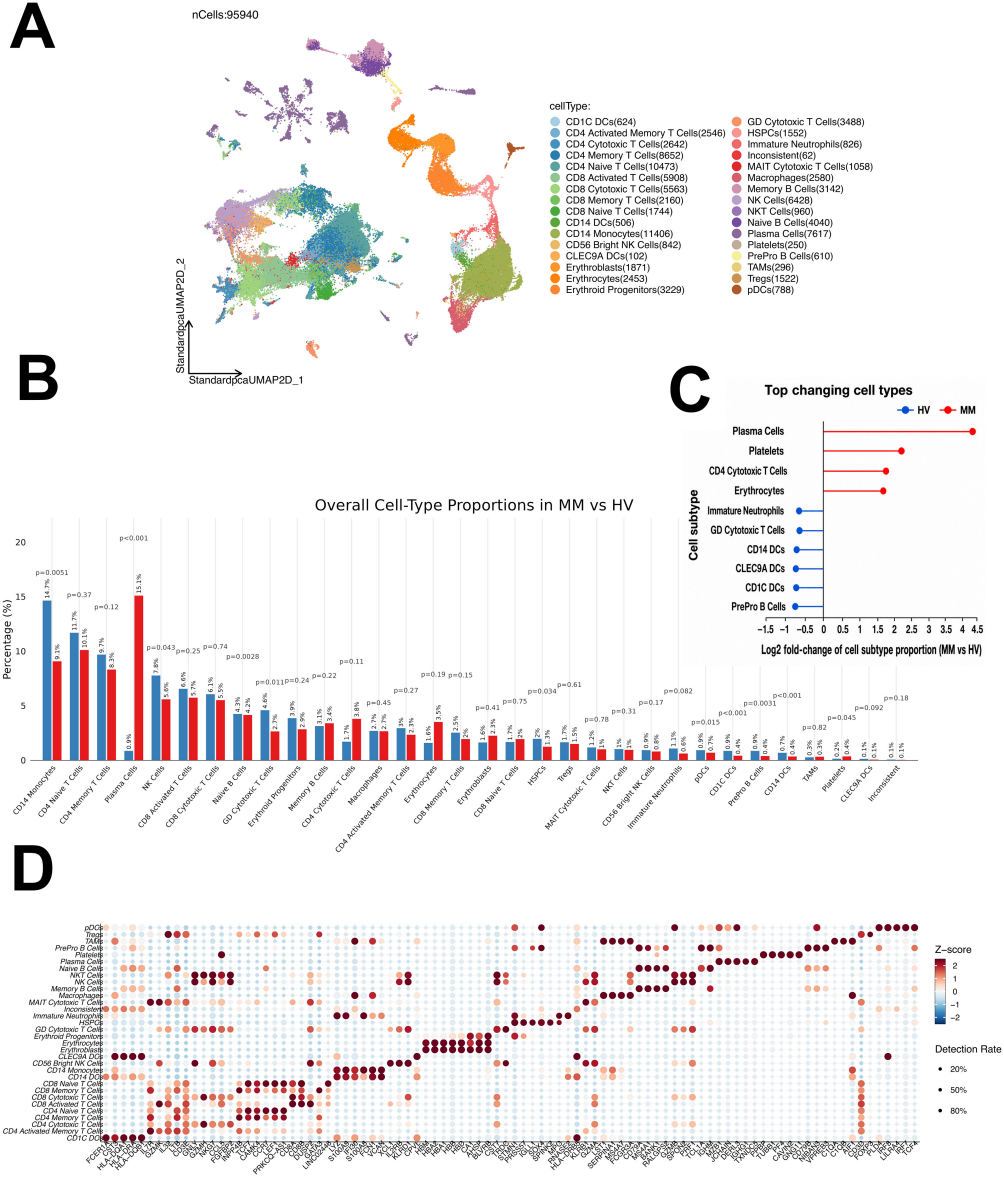
Global single-cell atlas and cellular composition remodeling of the MM bone marrow microenvironment. **(A)** UMAP visualization of 95,940 integrated bone marrow single cells colored by annotated cell identity. Thirty-two transcriptionally defined populations are resolved, spanning T/NK-cell subsets, B/plasma-cell subsets, dendritic cells, monocyte/macrophage lineages, erythroid and hematopoietic progenitor compartments, platelets, and minor or ambiguous clusters. Numbers in parentheses indicate the total number of cells assigned to each annotation after quality control, integration, dimensionality reduction, clustering, and marker-based annotation. **(B)** Cohort-level comparison of annotated cell-type composition between HV and MM. For each cohort, the proportion of a given cell population was calculated as the number of cells assigned to that population divided by the total number of cells analyzed within the same cohort. This panel provides a descriptive overview of broad marrow-ecosystem redistribution in MM. **(C)** Log2 fold-change of cell-subtype proportions comparing MM with HV. Positive values indicate proportional enrichment in MM, whereas negative values indicate proportional reduction in MM. The panel highlights major compositional shifts, including increased plasma cells, platelets, CD4 cytotoxic T cells, and erythrocytes, together with reduced PrePro B cells, dendritic-cell subsets, gamma-delta/GD cytotoxic T cells, and immature neutrophils. This effect-size panel should be interpreted as a ranked compositional summary. **(D)** Dot plot of canonical marker-gene expression used to support cell-type annotation. Dot color indicates Z-score-normalized average expression of each marker within the indicated cell type, and dot size indicates the detection rate, defined as the percentage of cells in that annotation with detectable marker expression.

We profiled 95,940 single cells from bone marrow samples of MM patients and HV. After quality control, integration, dimensionality reduction, and clustering, UMAP analysis resolved 32 transcriptionally distinct cell populations spanning T/NK-cell subsets, B/plasma-cell compartments, dendritic cells, monocyte/macrophage lineages, erythroid and hematopoietic progenitor populations, platelets, and minor cell populations (**Figure 1A**).

The relative distribution of these cell types differed substantially between HV and MM bone marrow (**Figure 1B**). In MM, plasma cells, platelets, CD4 cytotoxic T cells, and erythrocytes were among the most expanded populations, whereas PrePro B cells, dendritic-cell subsets, gamma-delta cytotoxic T cells, and immature neutrophils were reduced. Consistently, log2 fold-change analysis of cell-subtype proportions confirmed enrichment of plasma cells, platelets, CD4 cytotoxic T cells, and erythrocytes in MM, together with depletion of PrePro B cells, CD1C DCs, CLEC9A DCs, CD14 DCs, gamma-delta cytotoxic T cells, and immature neutrophils (**Figure 1C**). Canonical marker-gene expression further supported the cell-type annotations, including lineage-defining markers for T/NK cells, B/plasma cells, myeloid cells, dendritic cells, erythroid cells, progenitors, and platelets (**Figure 1D**). These data establish a global single-cell atlas of MM bone marrow and reveal extensive remodeling of immune, hematopoietic, and plasma-cell compartments.

### Global cell–cell communication networks are rewired in MM bone marrow

**Figure 2:**
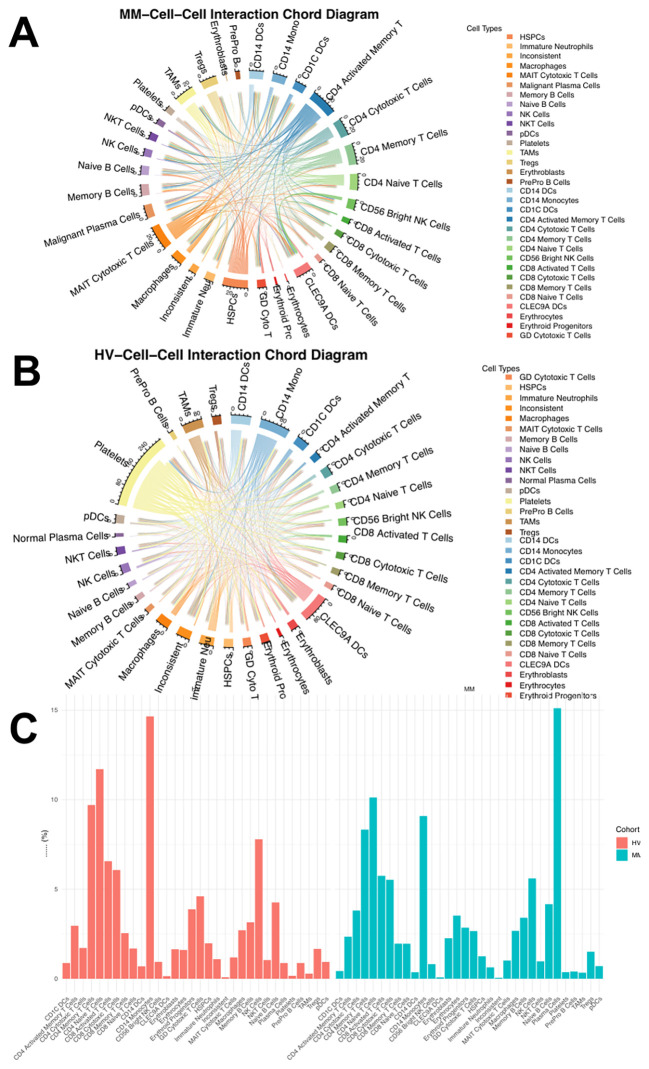
Global cell-cell communication rewiring in MM bone marrow. **(A)** CellChat-inferred chord diagram of predicted ligand-receptor communication among annotated cell populations in MM bone marrow. Sectors denote sender and receiver cell populations, and connecting ribbons represent inferred intercellular interactions. Ribbon density and width reflect the relative number or strength of predicted communication events. Malignant plasma cells are shown as a disease-associated plasma-cell compartment where indicated. **(B)** Chord diagram of inferred ligand-receptor communication among the same annotated cell populations in HV bone marrow. Comparison with panel A illustrates disease-associated rewiring of intercellular communication in MM, including altered participation of plasma-cell, myeloid, T/NK-cell, dendritic-cell, platelet, and progenitor compartments. Normal plasma cells are shown in the HV network where indicated. **(C)** Cohort-level relative abundance of annotated cell populations included in the communication analysis. Bars show cell-type percentages in HV and MM and provide compositional context for interpreting the CellChat-inferred network differences shown in **(A, B)**.

We next inferred intercellular communication networks to determine how cellular remodeling was accompanied by altered ligand–receptor interactions. CellChat-based analysis revealed broad rewiring of predicted cell–cell communication between HV and MM bone marrow ecosystems. In MM, chord diagrams showed densely interconnected communication involving malignant plasma cells, macrophage/TAM populations, T/NK-cell subsets, dendritic cells, and platelet-associated compartments, whereas HV marrow showed a more homeostatic communication architecture across immune and hematopoietic lineages (**Figures 2A, B**). Quantitative comparison further indicated that several disease-expanded or immunoregulatory compartments contributed prominently to the altered communication landscape in MM (**Figure 2C**).

Interaction-strength matrices provided an additional view of communication intensity. MM marrow contained stronger predicted communication involving malignant plasma cells, TAMs, macrophages, and platelet-related compartments, whereas HV marrow showed lower and more evenly distributed interaction intensities (**Supplementary Figures 1A, B**). These findings suggest that malignant transformation is associated not only with altered cellular composition but also with reorganization of local communication networks within the marrow microenvironment.

### T/NK-cell remodeling is heterogeneous and associated with subset-specific exhaustion and metabolic programs

**Figure 3:**
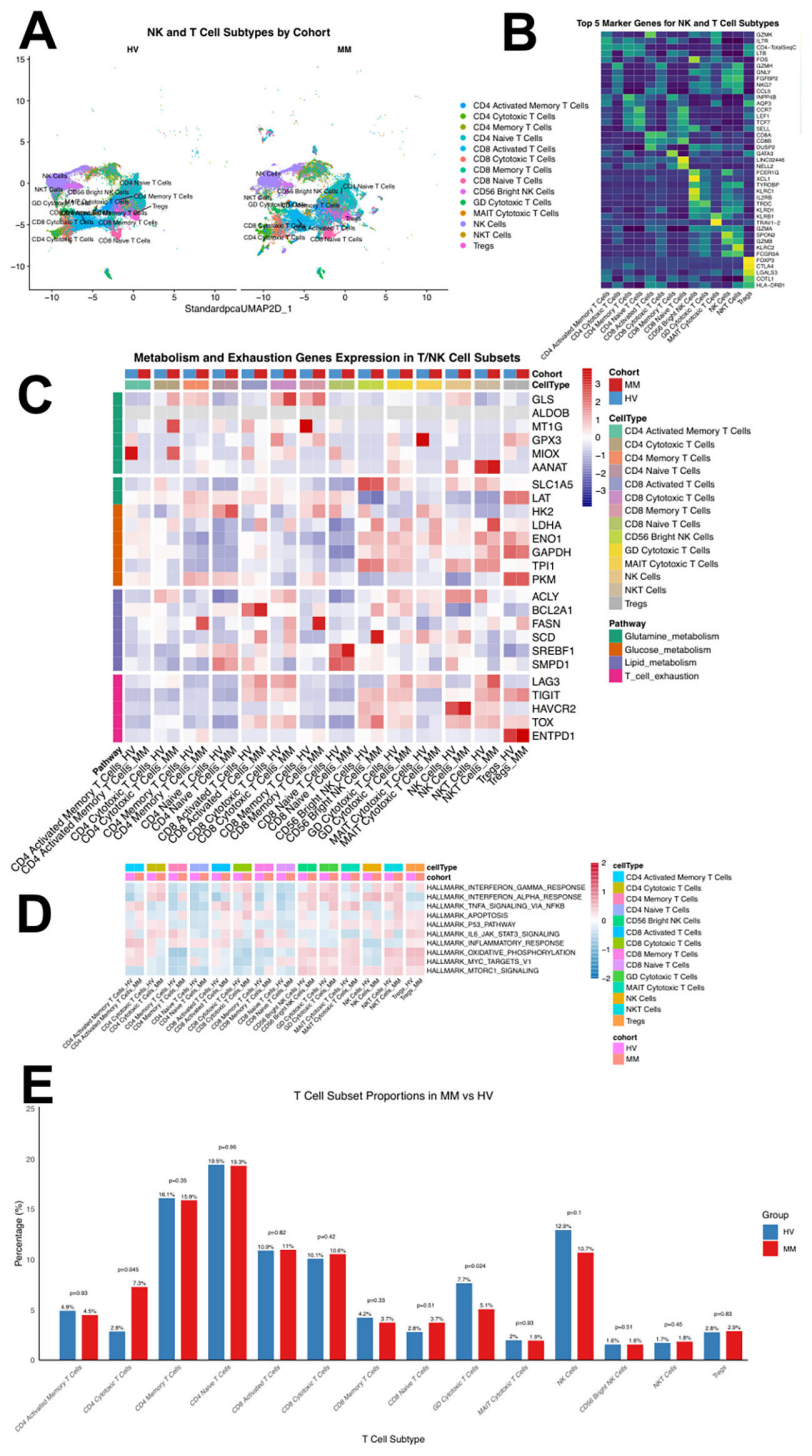
T/NK-cell subset remodeling and heterogeneous exhaustion-metabolic programs in MM. **(A)** UMAP visualization of reclustered T/NK-lineage cells stratified by cohort. Fourteen subsets are resolved: CD4 activated memory T cells, CD4 cytotoxic T cells, CD4 memory T cells, CD4 naive T cells, CD8 activated T cells, CD8 cytotoxic T cells, CD8 memory T cells, CD8 naive T cells, CD56 bright NK cells, GD cytotoxic T cells, MAIT cytotoxic T cells, NK cells, NKT cells, and Tregs. **(B)** Heatmap of top marker genes defining each T/NK subset. Average expression is summarized by annotated subset to support cell-state assignment, including cytotoxic programs, naive/memory T-cell programs, NK-lineage markers, MAIT/NKT-associated features, and regulatory T-cell markers. Color denotes scaled average expression. **(C)** Heatmap of selected metabolism- and exhaustion-associated genes across T/NK subtype-cohort combinations. Genes are grouped into glutamine metabolism, glucose metabolism, lipid metabolism, and exhaustion/checkpoint-associated programs. Columns represent paired subtype and cohort categories. The panel shows heterogeneous, subset-restricted remodeling rather than uniform up-regulation of glycolytic, lipid-metabolic, or exhaustion genes across all T/NK subsets. **(D)** Hallmark pathway activity heatmap for selected immune, inflammatory, and metabolic programs in T/NK subsets from HV and MM. Pathway scores were derived from GSVA or an equivalent gene-set scoring framework and visualized after scaling within the matrix. Displayed pathways include interferon responses, TNFalpha-NFkappaB signaling, IL6-JAK-STAT3 signaling, apoptosis, oxidative phosphorylation, MYC targets, and mTORC1 signaling. **(E)** Relative abundance of T/NK-cell subsets in HV and MM. Percentages were calculated within the T/NK compartment, and p values shown above bars summarize sample-level comparisons. The strongest descriptive changes include increased CD4 cytotoxic T cells and reduced GD cytotoxic or selected innate-like cytotoxic subsets, whereas Tregs remain essentially unchanged between HV and MM.

Reclustering of T/NK-lineage cells resolved 14 subsets spanning naïve, memory, cytotoxic, natural killer, NKT, MAIT, gamma-delta T-cell, and regulatory T-cell states (**Figure 3A**). Marker-gene analysis confirmed these annotations, with effector and cytotoxic populations characterized by GZMK, GZMH, GNLY, FGFBP2, NKG7, CCL5, and CD8A/CD8B, whereas naïve or memory populations showed enrichment of IL7R, CCR7, LEF1, and TCF7 (**Figure 3B**).

Expression profiling of metabolism- and exhaustion-related genes revealed heterogeneous remodeling across T/NK-cell subsets rather than uniform metabolic activation of the entire compartment. Glycolysis- and lipid-metabolism-associated genes, including HK2, LDHA, ACLY, FASN, SCD, and SREBF1, showed increased expression in selected MM T/NK subsets, whereas other subsets displayed modest, unchanged, or decreased expression patterns (**Figure 3C**). Similarly, exhaustion-associated genes, including LAG3, TIGIT, HAVCR2, TOX, and ENTPD1, were enriched in a subset-restricted manner, indicating an uneven exhaustion landscape within MM T/NK cells.

Pathway-level analysis further supported functional heterogeneity. Selected MM cytotoxic or activated T-cell states showed enrichment of inflammatory and cytokine-related programs, including IFN-gamma response, IL6–JAK–STAT3 signaling, and TNFα–NFκB signaling, whereas these pathways were not uniformly increased across all T/NK subsets (**Figure 3D**). Full Hallmark GSVA profiling similarly demonstrated subset-dependent immune and metabolic programs, with variable interferon-response, allograft-rejection, glycolysis, and IL6–JAK–STAT3 pathway activity across T/NK states (**Supplementary Figure 2**).

Relative abundance analysis further revealed compositional remodeling within the T/NK compartment. CD4 cytotoxic T cells increased from 2.8% in HV to 7.3% in MM (p = 0.045), whereas gamma-delta cytotoxic T cells decreased from 7.7% to 5.1% (p = 0.024). NK cells were numerically lower in MM (12.9% to 10.7%, p = 0.10). Tregs were essentially unchanged between HV and MM (2.8% vs. 2.9%, p = 0.83) and were not interpreted as increased (**Figure 3E**). Together, these findings indicate subset-specific T/NK-cell remodeling in MM, characterized by altered cytotoxic-cell composition, heterogeneous metabolic programs, and focal exhaustion-associated gene expression.

### Monocyte/macrophage lineages display TAM-associated inflammatory and immunometabolic remodeling

**Figure 4:**
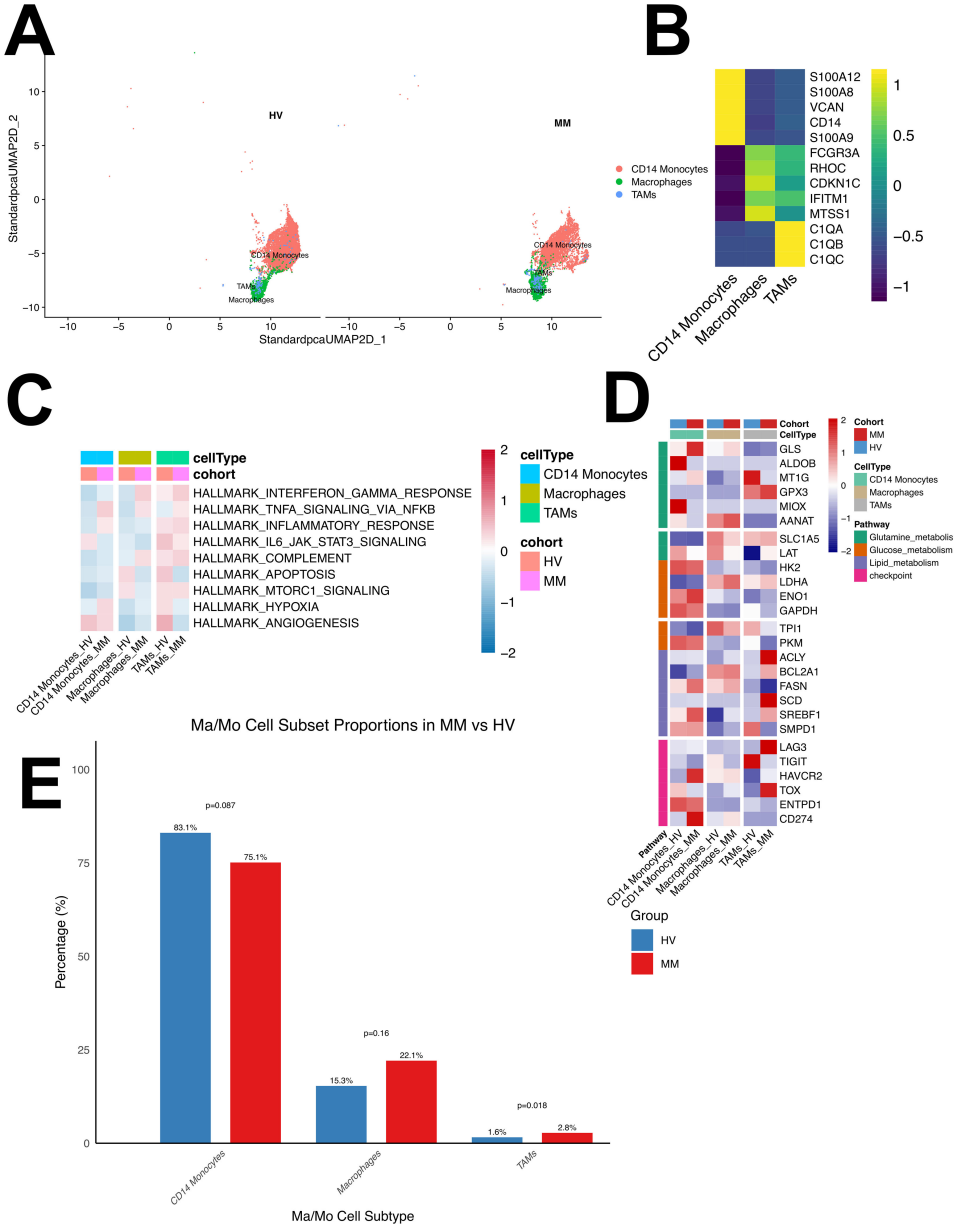
Monocyte/macrophage and TAM-associated inflammatory remodeling in MM. **(A)** UMAP visualization of reclustered monocyte/macrophage-lineage cells in HV and MM. Three transcriptionally distinct populations are shown: CD14 monocytes, macrophages, and tumor-associated macrophages (TAMs). **(B)** Heatmap of marker genes defining CD14 monocytes, macrophages, and TAMs. Cells from both HV and MM cohorts were included, and average expression was summarized by annotated cell type rather than by cohort. Representative markers include inflammatory monocyte markers such as S100A8, S100A9, VCAN, and CD14, macrophage-associated genes, and complement-related TAM markers such as C1QA, C1QB, and C1QC. **(C)** Heatmap of selected Hallmark pathway activities across CD14 monocytes, macrophages, and TAMs from HV and MM. Displayed programs include interferon-gamma response, TNFalpha-NFkappaB signaling, inflammatory response, IL6-JAK-STAT3 signaling, complement, apoptosis, mTORC1 signaling, hypoxia, and angiogenesis. The matrix highlights subset-dependent activation of inflammatory and stress-response programs rather than uniform myeloid activation. **(D)** Heatmap of metabolism- and checkpoint-associated genes across myeloid subtype-cohort combinations. Genes are grouped into glutamine metabolism, glucose metabolism, lipid metabolism, and checkpoint-related programs, including CD274. The panel supports a subset-specific interpretation in which selected metabolic and immune-regulatory genes are increased in particular MM myeloid states, especially macrophage/TAM-associated states. **(E)** Relative abundance of CD14 monocytes, macrophages, and TAMs in HV and MM. Percentages were calculated within the monocyte/macrophage compartment, and displayed p values indicate sample-level comparisons. TAMs show the clearest statistically supported increase in the plotted comparisons, whereas CD14 monocytes and macrophages should be interpreted as directional relative-abundance changes unless corrected significance is reported.

Reclustering of the monocyte/macrophage compartment identified three transcriptionally distinct populations: CD14 monocytes, macrophages, and TAMs (**Figure 4A**). Marker-gene expression supported these annotations, with CD14 monocytes enriched for S100A8, S100A9, VCAN, CD14, and S100A12; macrophages expressing FCGR3A, RHOC, CDKN1C, IFITM1, and MTSS1; and TAMs characterized by complement-associated genes including C1QA, C1QB, and C1QC (**Figure 4B**). The marker heatmap summarizes average expression across cells from both HV and MM cohorts and was used for cell-type annotation rather than cohort-specific comparison.

Pathway analysis revealed subset-dependent inflammatory remodeling in MM myeloid cells. IL6–JAK–STAT3, TNFα–NFκB, hypoxia-related, angiogenesis-associated, apoptosis, and mTORC1-associated programs were most evident in macrophage/TAM populations rather than uniformly activated across all myeloid subsets (**Figure 4C**). Consistently, metabolism- and checkpoint-related genes showed heterogeneous expression across CD14 monocytes, macrophages, and TAMs. Selected glycolytic and lipid-metabolism-associated genes, including ENO1, LDHA, FASN, and SCD, together with checkpoint-associated genes such as LAG3, TIGIT, HAVCR2, and CD274, showed increased expression in specific MM myeloid subsets, whereas other genes or subsets showed modest or unchanged expression patterns (**Figure 4D**). Full Hallmark pathway analysis further supported inflammatory activation and metabolic remodeling in macrophage/TAM states, including TNFα–NFκB, IL6–JAK–STAT3, and PI3K–AKT–mTOR-related activity, with altered complement pathway activity (**Supplementary Figure 3**).

Cell-proportion analysis showed a trend toward lower CD14 monocytes in MM (83.1% to 75.1%, p = 0.087), a numerical increase in macrophages (15.3% to 22.1%, p = 0.16), and a statistically supported increase in TAMs (1.6% to 2.8%, p = 0.018) (**Figure 4E**). These results support a shift from a CD14 monocyte-dominant myeloid compartment toward TAM-associated inflammatory and immunometabolic remodeling in MM marrow.

### B-cell and plasma-cell compartments show marked plasma-cell expansion and secretory-metabolic stress

**Figure 5:**
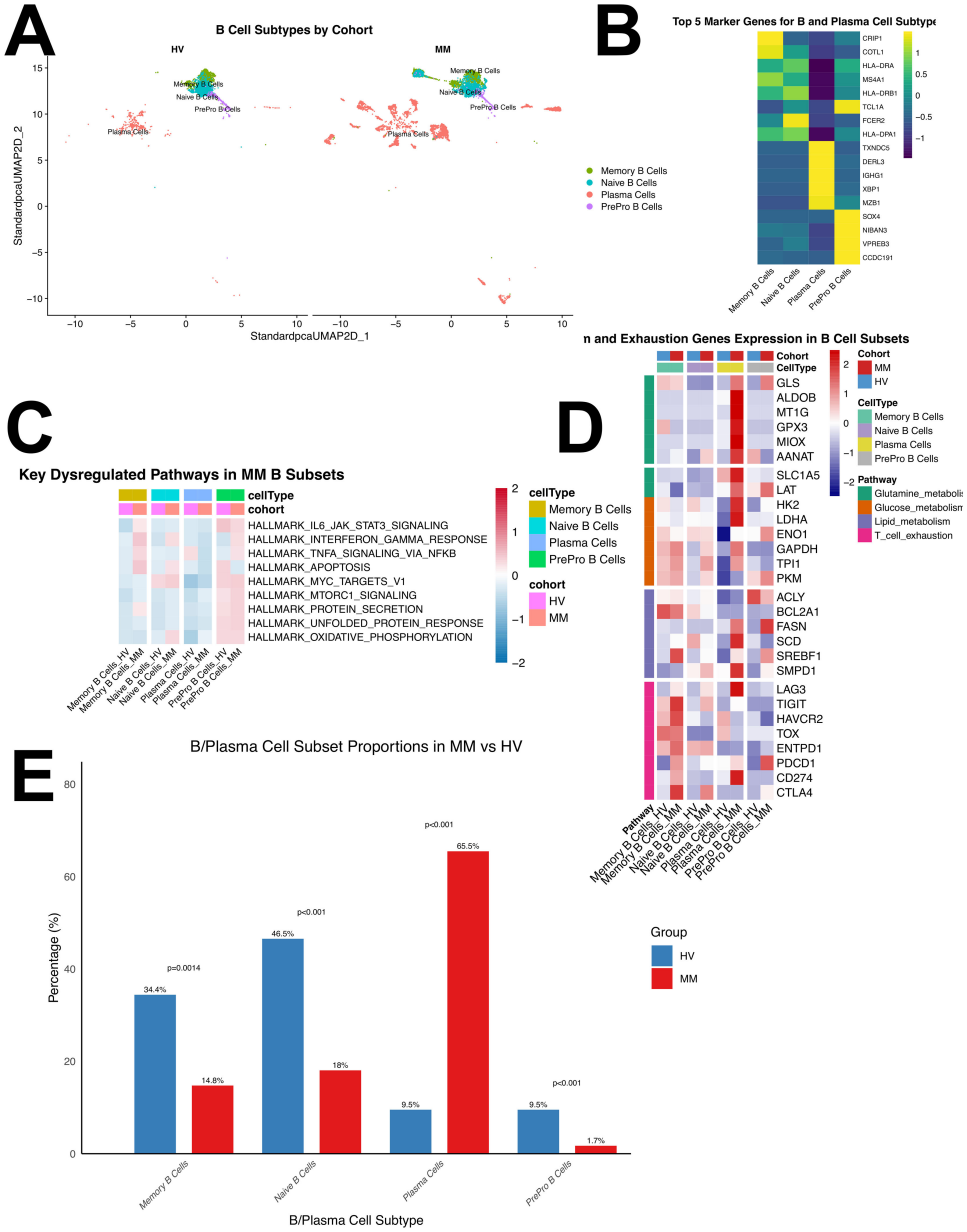
B-cell and plasma-cell compartment remodeling in MM. **(A)** UMAP visualization of reclustered B-lineage and plasma-cell populations, split by HV and MM. Four subsets are shown: memory B cells, naive B cells, plasma cells, and PrePro B cells. This subclustering separates expanded plasma-cell states from non-plasma B-cell compartments. **(B)** Heatmap of top marker genes supporting B/plasma-cell annotation. Representative markers include B-cell markers such as MS4A1 and TCL1A, plasma-cell and immunoglobulin-secretion-associated genes such as XBP1, MZB1, IGHG1, and DERL3, and PrePro B-cell-associated genes such as SOX4, NIBAN3, and VPREB3. Color indicates scaled average expression. **(C)** Selected Hallmark pathway activity heatmap in B/plasma-cell subsets from HV and MM. Displayed programs include IL6-JAK-STAT3 signaling, interferon-gamma response, TNFalpha-NFkappaB signaling, apoptosis, MYC targets, mTORC1 signaling, protein secretion, unfolded protein response (UPR), and oxidative phosphorylation. The enrichment of secretory and proteostatic stress programs in plasma cells is consistent with high immunoglobulin production and plasma-cell metabolic demand. **(D)** Heatmap of selected metabolism- and checkpoint/exhaustion-associated genes across B/plasma-cell subtype-cohort combinations. Genes are grouped into glutamine metabolism, glucose metabolism, lipid metabolism, and immune-regulatory programs. This panel links plasma-cell expansion to metabolic activation, lipid remodeling, and selected checkpoint-associated features. **(E)** Relative abundance of B/plasma-cell subsets in HV and MM. Percentages were calculated within the B/plasma-cell compartment, and p values shown above bars indicate sample-level comparisons. The most prominent change is marked plasma-cell expansion in MM, accompanied by proportional contraction of memory B cells, naive B cells, and PrePro B cells.

Four B-lineage populations were identified: memory B cells, naïve B cells, plasma cells, and PrePro B cells (**Figure 5A**). Marker-gene analysis confirmed lineage identity, with MS4A1, TCL1A, and FCER2 supporting B-cell states, and XBP1, MZB1, IGHG1, JCHAIN-related genes, and TXNDC5 supporting plasma-cell identity (**Figure 5B**).

Pathway analysis revealed pronounced activation of plasma-cell-associated secretory and metabolic stress programs in MM. MM plasma cells showed increased unfolded-protein response, protein secretion, oxidative phosphorylation, MYC targets, and mTORC1 signaling (**Figure 5C**). Metabolism- and checkpoint-related gene profiling further showed that MM plasma cells expressed targetable glucose- and glutamine-metabolic programs, including LDH-associated glycolytic/lactate genes such as HK2, LDHA, ENO1, GAPDH, TPI1, and PKM, together with glutamine-metabolism and glutamine-transport genes such as GLS, SLC1A5, and LAT (**Figure 5D**). These findings nominated LDH-dependent glycolytic/lactate metabolism and ASCT2/SLC1A5-mediated glutamine uptake as candidate functional vulnerabilities in the malignant plasma-cell compartment. Full Hallmark GSVA analysis confirmed enrichment of protein secretion, unfolded-protein response, MYC targets, oxidative phosphorylation, and mTORC1-related programs in MM plasma-cell states (**Supplementary Figure 4**).

Cell-proportion analysis demonstrated marked expansion of plasma cells in MM, increasing from 9.5% in HV to 65.5% in MM (p < 0.001). In contrast, memory B cells decreased from 34.4% to 14.8% (p = 0.0014), naïve B cells from 46.5% to 18.0% (p < 0.001), and PrePro B cells from 9.5% to 1.7% (p < 0.001) (**Figure 5E**). These data indicate that MM bone marrow is dominated by plasma-cell expansion coupled to protein-secretory stress and metabolic activation.

### Dendritic-cell remodeling reveals loss of conventional DC subsets and relative pDC enrichment

**Figure 6:**
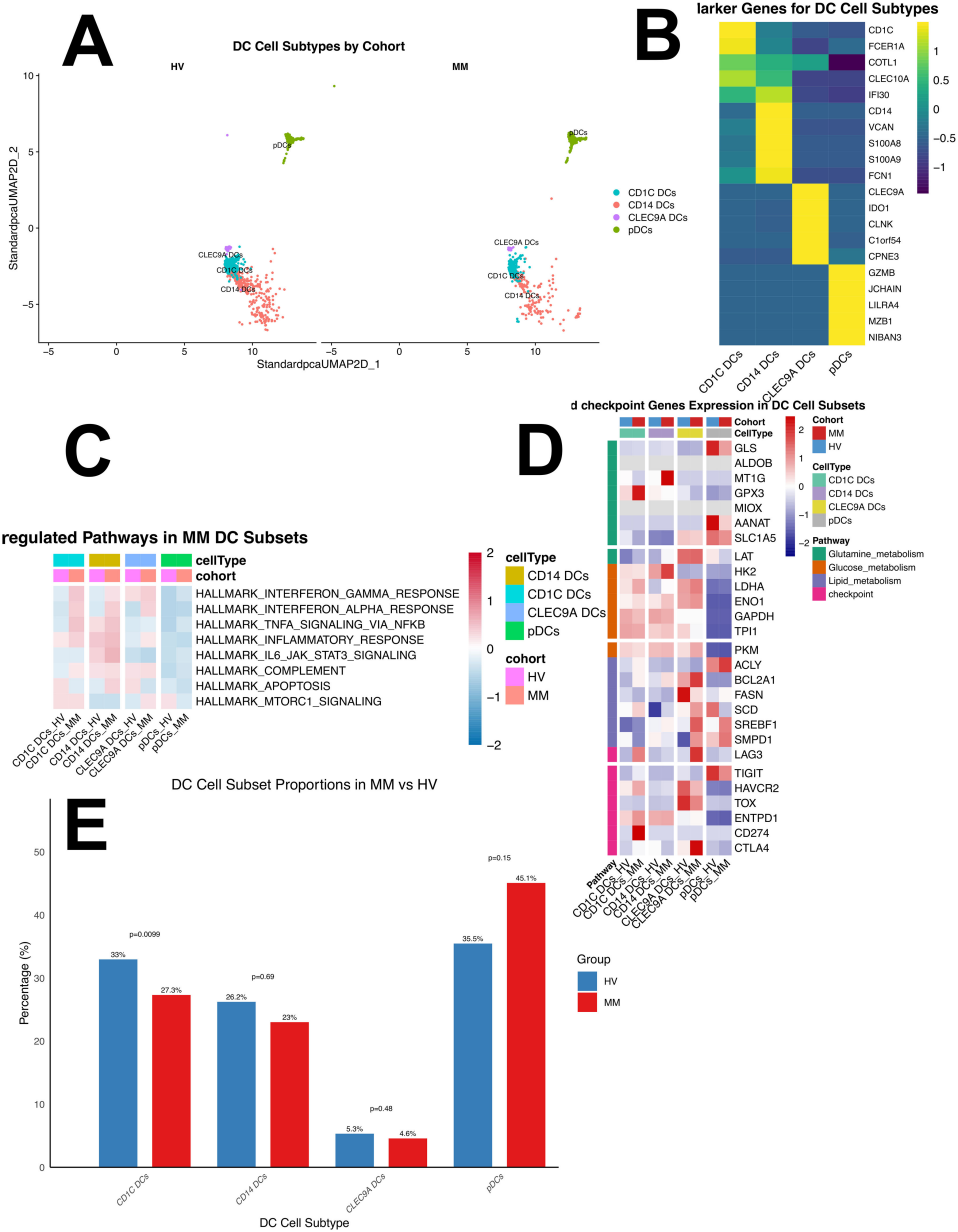
Dendritic-cell subset remodeling in MM. **(A)** UMAP visualization of reclustered dendritic-cell populations in HV and MM. Four subsets are resolved: CD1C DCs, CD14 DCs, CLEC9A DCs, and plasmacytoid DCs (pDCs). **(B)** Heatmap of top marker genes for dendritic-cell subset annotation. Representative markers include CD1C, FCER1A, CLEC10A, and IFI30 for CD1C DCs; CD14, VCAN, S100A8, S100A9, and FCN1 for CD14 DCs; CLEC9A, IDO1, CLNK, C1orf54, and CPNE3 for CLEC9A DCs; and GZMB, JCHAIN, LILRA4, MZB1, and NIBAN3 for pDCs. Color reflects scaled average expression. **(C)** Selected Hallmark pathway activities in dendritic-cell subsets from HV and MM. Displayed pathways include interferon-alpha and interferon-gamma responses, TNFalpha-NFkappaB signaling, inflammatory response, IL6-JAK-STAT3 signaling, complement, apoptosis, and mTORC1 signaling. These pathways summarize immune activation, antigen-presenting function, and stress-response remodeling within the DC compartment. **(D)** Heatmap of metabolism- and checkpoint-associated genes across DC subtype-cohort combinations. Genes are grouped into glutamine metabolism, glucose metabolism, lipid metabolism, and checkpoint-related categories. The panel supports a subset-specific rather than uniform interpretation of metabolic and immune-regulatory remodeling in dendritic cells. **(E)** Relative abundance of dendritic-cell subsets in HV and MM. Percentages were calculated within the DC compartment, and p values shown above bars indicate sample-level comparisons. CD1C DCs show a statistically supported reduction in MM, whereas changes in CD14 DCs, CLEC9A DCs, and pDCs should be interpreted cautiously according to the displayed p values.

Subclustering of dendritic cells identified four transcriptionally distinct subsets: CD1C DCs, CD14 DCs, CLEC9A DCs, and plasmacytoid DCs (pDCs) (**Figure 6A**). Marker-gene expression confirmed these identities, including CD1C, FCER1A, CLEC10A, and IFI30 for CD1C DCs; CD14, VCAN, S100A8, S100A9, and FCN1 for CD14 DCs; CLEC9A, IDO1, CLNK, and CPNE3 for CLEC9A DCs; and GZMB, JCHAIN, LILRA4, MZB1, and NIBAN3 for pDCs (**Figure 6B**).

Pathway-level analysis demonstrated subset-dependent immune and metabolic alterations among DC populations. Selected MM DC subsets showed altered interferon-response, TNFα–NFκB, inflammatory-response, IL6–JAK–STAT3, apoptosis, and mTORC1-associated programs (**Figure 6C**). Metabolism- and checkpoint-related gene profiling showed variable expression of glucose-metabolism, lipid-metabolism, glutamine-metabolism, and checkpoint-associated genes across DC subsets, indicating subset-dependent remodeling rather than uniform activation (**Figure 6D**). Full Hallmark pathway analysis further demonstrated broad variation in inflammatory, interferon, glycolytic, and TGF-beta-related programs across DC states (**Supplementary Figure 5**).

Cell-proportion analysis showed a reduction in CD1C DCs in MM (33.0% to 27.3%, p = 0.0099), whereas CD14 DCs (26.2% to 23.0%, p = 0.69) and CLEC9A DCs (5.3% to 4.6%, p = 0.48) changed modestly. pDCs were numerically increased in MM (35.5% to 45.1%, p = 0.15), although this did not reach statistical significance (**Figure 6E**). These findings suggest reduction of selected conventional DC populations and a relative shift toward pDC-enriched immunoregulatory states in MM marrow.

### HSPC and blood-lineage compartments show lineage-skewing trends and stress-associated pathway programs

**Figure 7:**
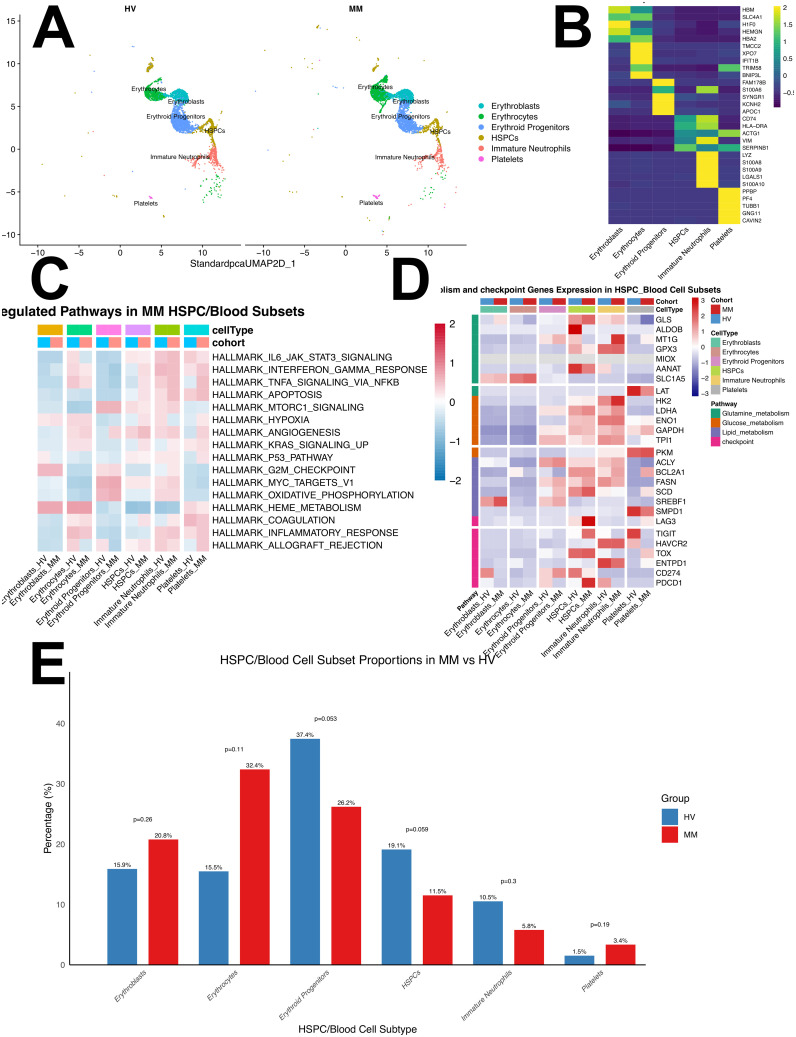
HSPC and blood-lineage compartment remodeling in MM. **(A)** UMAP visualization of reclustered HSPC/blood-lineage populations in HV and MM. Six subsets are shown: erythroblasts, erythrocytes, erythroid progenitors, hematopoietic stem/progenitor cells (HSPCs), immature neutrophils, and platelets. **(B)** Heatmap of marker genes defining the HSPC/blood-lineage subsets. Representative genes include hemoglobin and erythroid genes for erythroblasts and erythrocytes, progenitor-associated markers for HSPCs and erythroid progenitors, neutrophil-associated inflammatory genes such as LYZ, S100A8, and S100A9, and platelet-associated genes including PPBP, PF4, TUBB1, GNG11, and CAVIN2. Color represents scaled average expression. **(C)** Selected Hallmark pathway activity heatmap across HSPC/blood-lineage subtype-cohort combinations. Displayed programs include IL6-JAK-STAT3 signaling, interferon-gamma response, TNFalpha-NFkappaB signaling, apoptosis, mTORC1 signaling, hypoxia, angiogenesis, KRAS signaling, p53 pathway, G2M checkpoint, MYC targets, oxidative phosphorylation, heme metabolism, coagulation, inflammatory response, and allograft rejection. These pathways summarize hematopoietic stress, proliferative signaling, inflammatory tone, and erythroid/platelet-associated biology. **(D)** Heatmap of selected metabolism- and checkpoint-associated genes in HSPC/blood-lineage subsets. Genes are grouped into glutamine metabolism, glucose metabolism, lipid metabolism, and checkpoint-related programs. The panel explores whether metabolic and immune-regulatory features extend beyond classical immune compartments. **(E)** Relative abundance of erythroblasts, erythrocytes, erythroid progenitors, HSPCs, immature neutrophils, and platelets in HV and MM. Percentages were calculated within the HSPC/blood-lineage compartment, and p values are shown for sample-level comparisons. Because the displayed comparisons do not reach conventional significance, these findings should be described as directional or trend-level compositional changes rather than definitive expansion or depletion.

Reclustering of HSPC and blood-lineage compartments identified erythroblasts, erythrocytes, erythroid progenitors, HSPCs, immature neutrophils, and platelets (**Figure 7A**). Marker-gene analysis supported lineage annotation, including erythroid/hemoglobin-associated genes for erythroblasts and erythrocytes, progenitor-associated genes for HSPCs and erythroid progenitors, neutrophil-related genes such as LYZ, S100A8, and S100A9, and platelet-associated genes including PPBP, PF4, TUBB1, GNG11, and CAVIN2 (**Figure 7B**).

Pathway analysis showed altered inflammatory, metabolic, proliferative, coagulation, heme-metabolism, and stress-response programs across HSPC/blood-lineage subsets (**Figure 7C**). Metabolism- and checkpoint-related gene profiling further revealed variable glucose-, lipid-, glutamine-metabolism, and checkpoint-associated gene expression across erythroid, progenitor, neutrophil, and platelet-related states (**Figure 7D**). Full Hallmark GSVA analysis showed lineage-dependent changes involving IL6–JAK–STAT3 signaling, TNFα–NFκB signaling, MYC targets, oxidative phosphorylation, heme metabolism, coagulation, G2M checkpoint, and inflammatory-response programs (**Supplementary Figure 6**).

Cell-proportion analysis showed numerical increases in erythroblasts (15.9% to 20.8%, p = 0.26), erythrocytes (15.5% to 32.4%, p = 0.11), and platelets (1.5% to 3.4%, p = 0.19), alongside lower erythroid progenitors (37.4% to 26.2%, p = 0.053), HSPCs (19.1% to 11.5%, p = 0.059), and immature neutrophils (10.5% to 5.8%, p = 0.30) in MM (**Figure 7E**). Although these changes did not uniformly reach statistical significance, they suggest lineage-skewing trends and stress-associated hematopoietic remodeling in the MM marrow microenvironment.

### Disease-associated communication circuits and clinical validation converge on checkpoint and IL-6-associated remodeling

**Figure 8:**
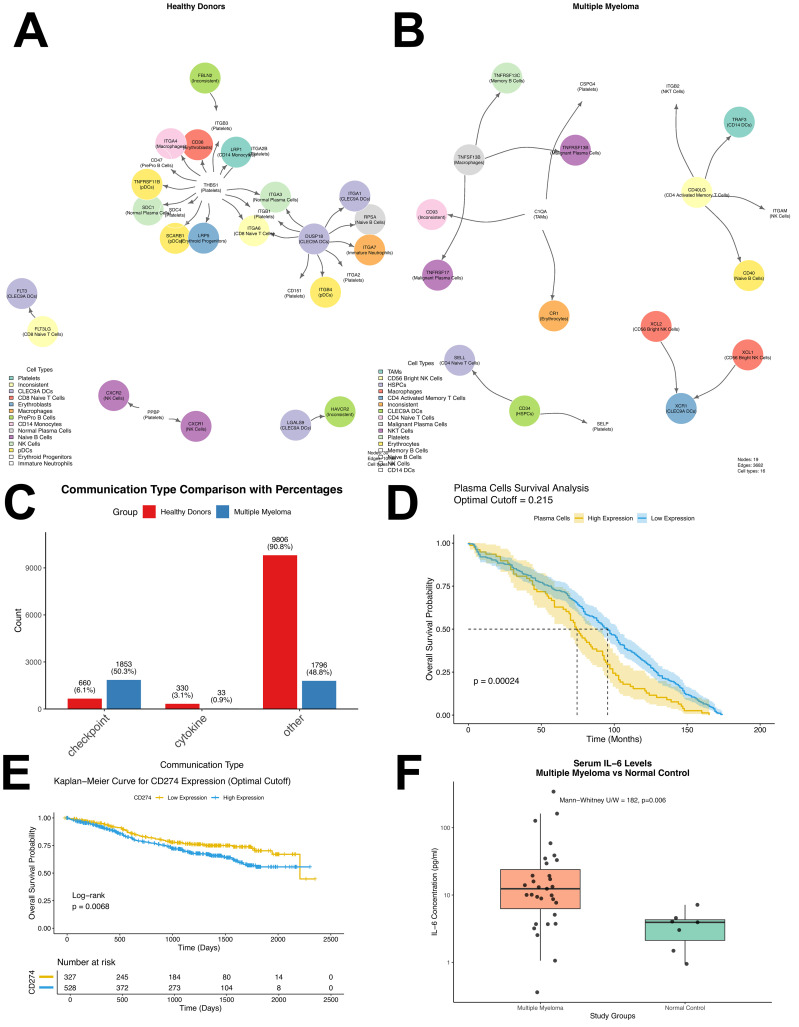
Disease-associated communication circuits and clinical validation of an IL6-associated immunometabolic axis. **(A)** Representative ligand-receptor communication network in healthy donor marrow. Nodes correspond to ligand or receptor genes annotated by their source or target cell type, and edges indicate inferred communication relationships. The healthy network provides a baseline reference for marrow intercellular signaling. **(B)** Representative ligand-receptor communication network in MM marrow. Disease-associated nodes include malignant plasma-cell, TAM/macrophage, T/NK-cell, dendritic-cell, platelet, and hematopoietic compartments. Highlighted axes include plasma-cell-associated TNFRSF17/TNFRSF13-family signaling, macrophage/TAM-associated inflammatory mediators, and T/NK/DC-associated communication modules, supporting the concept of a rewired tumor-supportive signaling network. **(C)** Quantitative comparison of inferred communication-type composition between healthy donors and MM. Interactions are classified as checkpoint, cytokine, or other communication categories, and bars show interaction counts and percentages within each cohort. In MM, checkpoint-classified interactions increase to 50.3%, with other interactions accounting for 48.8% and cytokine-classified interactions accounting for 0.9%, indicating a shift toward checkpoint-enriched and non-canonical communication programs. **(D)** Kaplan-Meier survival analysis stratifying patients by plasma-cell-associated feature level. Patients were divided into high and low groups using the indicated cutoff, and overall survival was compared using the log-rank test. This panel evaluates the clinical relevance of plasma-cell burden or plasma-cell-associated transcriptional activity. **(E)** Kaplan-Meier survival analysis stratifying patients by CD274 (PD-L1) expression. Patients were divided into high and low expression groups according to the indicated cutoff, and survival curves were compared by log-rank test. The association supports the clinical relevance of immune-checkpoint engagement within the MM immunometabolic network. **(F)** Independent clinical validation of serum IL-6 levels. Serum IL-6 concentrations were compared between MM patients and normal controls using a nonparametric Mann-Whitney U test. These data were obtained from an independent clinical cohort rather than matched serum samples from the public scRNA-seq dataset and support systemic activation of the IL6-associated inflammatory axis inferred from transcriptomic and communication analyses.

We next integrated ligand–receptor networks with communication-category analysis to identify disease-associated signaling features. Representative communication networks revealed distinct ligand–receptor architectures in HV and MM. In HV, communication involved homeostatic interactions among platelets, dendritic cells, naïve/memory lymphoid populations, normal plasma cells, and myeloid cells. In MM, the network highlighted disease-associated communication involving TAMs, macrophages, malignant plasma cells, CD56 bright NK cells, CD4 activated memory T cells, dendritic cells, platelets, and B-cell subsets (**Figures 8A, B**). Prominent MM-associated ligand–receptor features included C1QA, TNFSF13B, CD40LG, XCL1/XCL2–XCR1, TNFRSF17, TNFRSF13B, TNFRSF13C, CD40, and integrin/adhesion-related signals, supporting extensive rewiring of immune, plasma-cell, and myeloid communication. Communication type-stratified chord diagrams further supported redistribution of checkpoint, cytokine, and other signaling categories between HV and MM (**Supplementary Figures 7A, B**).

Communication-type quantification showed a marked redistribution of predicted signaling categories. In healthy donor marrow, “other” interactions accounted for 90.8% of predicted interactions, checkpoint interactions for 6.1%, and cytokine interactions for 3.1%. In MM, checkpoint interactions increased to 50.3%, with “other” interactions accounting for 48.8% and cytokine-classified interactions for 0.9% (**Figure 8C**). These findings indicate that MM is associated with a shift toward checkpoint-enriched and non-canonical communication programs linking malignant plasma cells, TAMs, and immune effector populations.

We then evaluated the clinical relevance of these network-level features. Patients with high plasma-cell-associated feature expression had inferior overall survival compared with those in the low-expression group (median overall survival 3.93 vs. 12.4 months; log-rank p = 0.00024) (**Figure 8D**). High CD274 expression was also associated with worse overall survival (log-rank p = 0.0068) (**Figure 8E**). In an independent clinical validation cohort, serum IL-6 concentrations were significantly higher in MM patients than in normal controls (Mann–Whitney U/W = 182, p = 0.006) (**Figure 8F**). Together, these data link plasma-cell expansion, checkpoint activation, and IL-6-associated inflammation to clinically adverse disease features.

### Biological and functional validation supports an immunometabolic vulnerability in MM

**Figure 9:**
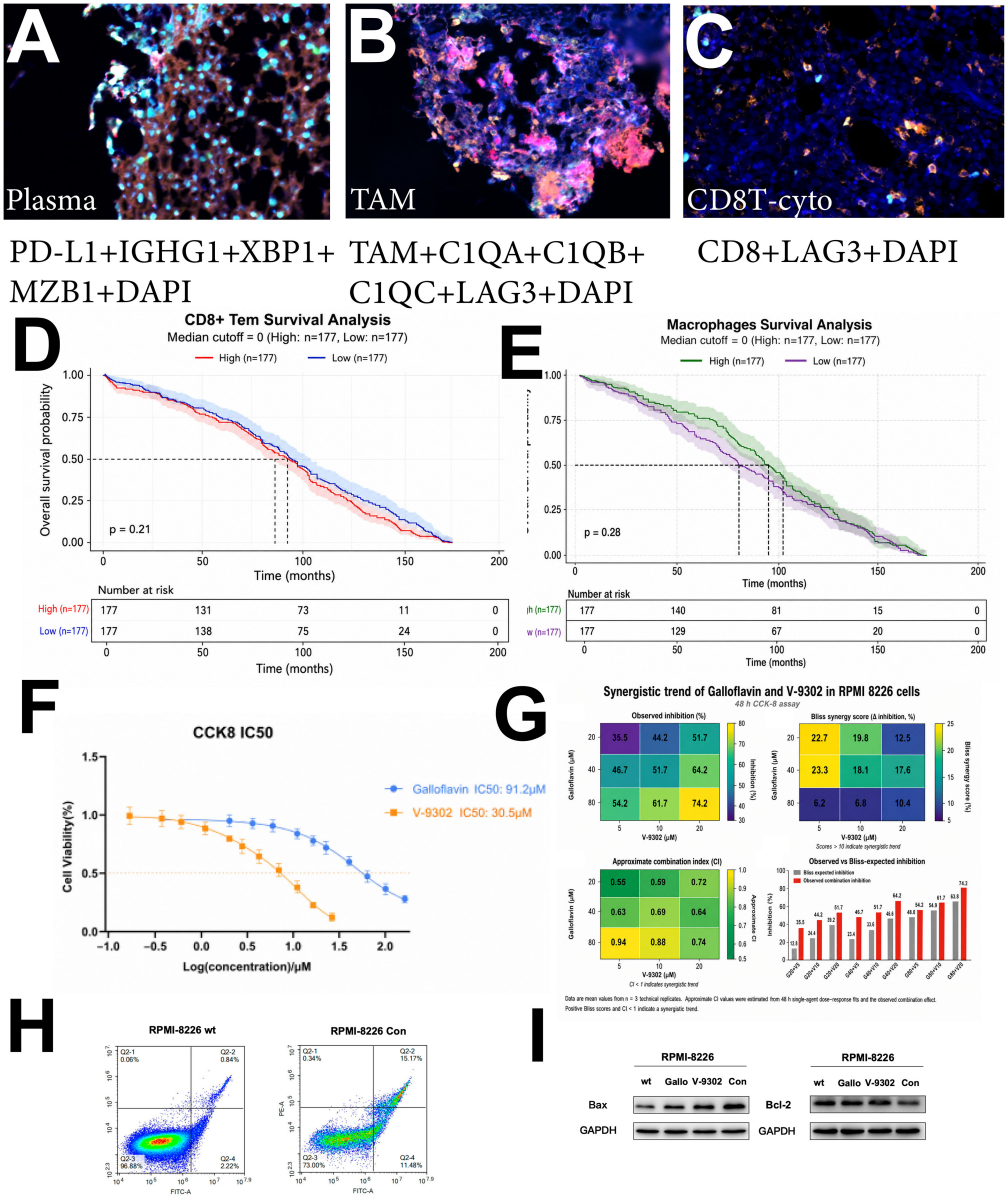
Biological and functional validation of the MM immunometabolic circuit. **(A)** Multiplex immunofluorescence validation of PD-L1-positive plasma cells expressing IGHG1, XBP1, and MZB1. Nuclei are counterstained with DAPI. **(B)** Multiplex immunofluorescence validation of C1QA/C1QB/C1QC-positive TAMs with LAG3 expression. Nuclei are counterstained with DAPI. **(C)** CD8-positive/LAG3-positive cytotoxic T cells in MM tissue sections. Nuclei are counterstained with DAPI. Together, panels A-C provide spatial and protein-level support for the coexistence of plasma-cell, TAM, and dysfunctional T-cell components implicated by the single-cell and communication analyses. **(D)** Kaplan-Meier survival analysis of CD8-positive effector-memory T-cell-associated features. Patients were stratified into high and low groups according to the indicated cutoff, and p values were calculated by log-rank test. **(E)** Kaplan-Meier survival analysis of macrophage-associated features. Patients were stratified into high and low groups according to the indicated cutoff, and p values were calculated by log-rank test. **(D, E)** assess whether individual immune-cell-associated features alone capture clinical outcome heterogeneity. **(F)** CCK-8 dose-response curves and IC50 estimation for galloflavin, an LDH inhibitor targeting glycolytic/lactate metabolism, and V-9302, an ASCT2/SLC1A5 glutamine-transport inhibitor, in RPMI-8226 cells. Viability was normalized to vehicle-treated controls, and fitted curves were used to estimate inhibitory potency. **(G)** Drug-combination analysis of galloflavin and V-9302 integrating observed inhibition, Bliss synergy score, approximate combination index (CI), and observed-versus-Bliss-expected inhibition. Positive Bliss synergy scores, CI values below 1, and observed inhibition exceeding Bliss-expected inhibition support a cooperative metabolic vulnerability. NT denotes not tested. Data are mean values from n = 3 technical replicates, and approximate CI values were estimated from 48-h single-agent dose-response fits and the observed combination effect. **(H)** Apoptosis-related validation after metabolic perturbation. Western blotting assesses Bax and Bcl-2 protein levels with GAPDH as loading control, **(I)** Flow-cytometry apoptosis analysis quantifies apoptotic fractions. Together with panels **(F–H)**, these data provide functional support for targeting glycolytic/lactate and glutaminedependent metabolic dependencies inferred from the single-cell immunometabolic landscape.

To validate the cellular and functional relevance of the inferred immunometabolic circuit, we moved the expanded biological validation data into the main figures as **Figure 9**. Multiplex immunofluorescence confirmed the presence of PD-L1-positive plasma cells, C1QA/C1QB/C1QC-positive TAMs with LAG3 expression, and CD8-positive/LAG3-positive cytotoxic T cells in MM tissue sections (**Figures 9A–C**). These findings provide tissue-level support for the coexistence of malignant plasma-cell, TAM, and dysfunctional T-cell compartments predicted by the single-cell and communication analyses.

Survival analysis of immune-cell-associated features showed that CD8-positive effector-memory T-cell and macrophage-associated abundance alone did not significantly stratify overall survival (p = 0.28 and p = 0.21, respectively) (**Figures 9D, E**). These observations suggest that prognosis may be better captured by integrated immunometabolic and checkpoint-associated network features than by isolated immune-cell frequencies alone.

We next tested whether the metabolic dependencies inferred from the MM plasma-cell transcriptional program could be functionally targeted in myeloma cells. Because MM plasma cells showed activation of LDH-associated glycolytic/lactate genes and ASCT2/SLC1A5-GLS-linked glutamine-metabolic programs (**Figure 5D**), we selected two pathway-matched inhibitors for validation. Galloflavin was used to inhibit LDH-dependent pyruvate-to-lactate conversion, whereas V-9302 was used to block ASCT2/SLC1A5-mediated glutamine transport. Both compounds reduced RPMI-8226 cell viability in a dose-dependent manner, with estimated IC50 values of approximately 91.2 μM and 30.5 μM, respectively (**Figure 9F**). Drug-combination analysis further demonstrated synergistic anti-myeloma activity. The combination panel integrated four complementary readouts: observed inhibition, Bliss synergy score, approximate combination index, and observed versus Bliss-expected inhibition. Positive Bliss synergy scores, combination-index values below 1, and observed inhibition exceeding Bliss-expected inhibition together supported a synergistic interaction between galloflavin and V-9302 (**Figure 9G**).

Additional functional validation further supported this metabolic vulnerability. Time-course viability assays showed progressive suppression of RPMI-8226 viability after metabolic inhibitor treatment over 24, 48, and 72 h (**Figure 9H**). Apoptosis-related validation demonstrated Bax/Bcl-2 modulation and increased apoptotic fractions by western blotting and flow cytometry, indicating activation of apoptotic programs following metabolic perturbation (**Figure 9I**). Together, these results provide convergent evidence that simultaneous targeting of glycolytic and glutamine-dependent metabolic pathways produces synergistic anti-myeloma activity and functionally validates metabolic vulnerabilities inferred from single-cell immunometabolic profiling.

### Integrated pathway summary supports an IL6-centered immunometabolic circuit in MM

Finally, we integrated pathway-level, cellular, communication-network, clinical, and experimental findings into a unified immunometabolic framework. An integrated Hallmark pathway dot plot summarized recurrent pathway remodeling across T/NK cells, monocyte/macrophage/TAM populations, B/plasma-cell compartments, dendritic cells, and HSPC/blood-lineage populations (**Supplementary Figure 8A**). Recurrently altered programs included IL6–JAK–STAT3 signaling, TNFα–NFκB signaling, oxidative phosphorylation, mTORC1 signaling, protein secretion/unfolded protein response, hypoxia, angiogenesis, and immune-checkpoint/exhaustion-associated pathways.

Based on these convergent findings, we constructed a conceptual model of an IL6-centered immunometabolic circuit in MM (**Supplementary Figure 8B**). In this model, malignant plasma cells, TAMs, and dysfunctional T cells are linked through ligand–receptor signaling, inflammatory cytokine programs, metabolic stress, and checkpoint activation. Plasma-cell-associated molecules such as TNFRSF17 and SDC1, macrophage/TAM-associated inflammatory mediators such as IL-6, and checkpoint/exhaustion molecules including PD-L1, LAG3, TIGIT, and HAVCR2 collectively define a self-reinforcing immunometabolic niche. This circuit provides a mechanistic framework connecting cellular remodeling, IL6–JAK–STAT3 activation, TAM–plasma cell–T-cell communication, immune suppression, adverse clinical features, and therapeutic vulnerability to metabolic intervention.

## Discussion

This study delineates an immunometabolic communication landscape of the MM bone marrow microenvironment at single-cell resolution. By integrating cellular composition, pathway activity, ligand–receptor communication, clinical validation, spatial immunophenotyping, and metabolic perturbation assays, we identify a disease-associated circuit linking malignant plasma cells, TAMs, and dysfunctional T cells. Rather than representing independent cellular abnormalities, these alterations converge on a coordinated axis characterized by IL6–JAK–STAT3 activation, checkpoint-enriched communication, plasma-cell metabolic stress, myeloid inflammatory remodeling, and therapeutically targetable metabolic dependencies.

### An IL6-centered immunometabolic state emerges from coordinated cellular remodeling

The single-cell atlas revealed broad remodeling of the MM marrow ecosystem, with expansion of plasma cells, platelets, CD4 cytotoxic T cells, and erythroid-lineage cells, together with depletion of multiple antigen-presenting and immune-effector populations (**Figure 1**). These changes are consistent with the concept that MM progression is not only a plasma-cell-intrinsic process but also a systems-level remodeling of the marrow niche. Prior studies have shown that MM is accompanied by profound immune microenvironmental alterations and malignant cell-mediated immunosuppression (2, 5). Our data extend this concept by showing that compositional changes are coupled to metabolic and communication-level reprogramming across multiple marrow compartments.

The integrated pathway summary further supports this view. Across T/NK cells, monocyte/macrophage/TAM populations, B/plasma-cell compartments, dendritic cells, and HSPC/blood-lineage subsets, recurrently altered programs included IL6–JAK–STAT3 signaling, TNFα–NFκB signaling, oxidative phosphorylation, mTORC1 signaling, unfolded protein response/protein secretion, hypoxia, and angiogenesis (**Supplementary Figure 8A**). These pathways are not uniformly activated in every cell type; rather, they define a compartment-specific but coordinated disease state. The MM microenvironment therefore appears to reshape each compartment according to lineage identity while converging on shared inflammatory and metabolic stress axes.

Among these programs, IL6–JAK–STAT3 signaling is particularly relevant. IL-6 has long been implicated in myeloma-cell growth, survival, inflammatory remodeling, and disease severity, and JAK–STAT3 signaling is a central downstream pathway linking cytokine stimulation to tumor survival and immune regulation (8–10). In our study, elevated serum IL-6 in an independent clinical cohort, enrichment of IL6–JAK–STAT3-related programs in single-cell analyses, and poor clinical outcomes associated with plasma-cell and CD274 features support an IL6-centered interpretation of MM immunometabolism (**Figures 8D–F**). The IL-6 serum validation should be interpreted as an independent clinical validation layer rather than matched patient-level validation of the public scRNA-seq cohort, but its consistency with transcriptomic and communication findings strengthens the biological plausibility of this axis.

### Immune dysfunction in MM is subset-specific rather than uniformly exhausted

A major implication of the T/NK-cell analysis is that immune dysfunction in MM should not be reduced to a single exhausted T-cell state. Reclustering revealed multiple T/NK subsets with distinct compositional, metabolic, and checkpoint-associated features (**Figure 3**). Some subsets showed increased expression of glycolysis- or lipid-metabolism-associated genes, whereas others showed unchanged or reduced expression. Exhaustion-associated markers such as LAG3, TIGIT, HAVCR2, TOX, and ENTPD1 were also enriched in a subset-restricted manner rather than uniformly across the T/NK compartment. Averaged lineage-level statements can obscure this functionally important heterogeneity.

The proportional data further support a selective pattern of T/NK remodeling. CD4 cytotoxic T cells increased, gamma-delta cytotoxic T cells decreased, NK cells showed a numerical reduction, and Tregs remained essentially unchanged (**Figure 3E**). Thus, the most relevant immune changes are not a generalized increase in immunosuppressive Tregs but rather redistribution and functional remodeling of cytotoxic and innate-like effector populations. This observation is consistent with the view that MM immune escape may result from altered effector-cell quality, checkpoint engagement, and metabolic stress rather than from a simple shift in one regulatory population. It also aligns with prior evidence that T-cell exhaustion contributes to myeloma immune escape and therapeutic resistance (7).

### TAM-associated inflammatory remodeling links myeloid activation to checkpoint and metabolic programs

The myeloid compartment showed a complementary pattern of disease-associated remodeling. CD14 monocytes, macrophages, and TAMs formed transcriptionally distinct subsets, with TAMs characterized by C1QA, C1QB, and C1QC (**Figures 4A, B**). Pathway and gene-expression analyses indicated that inflammatory and metabolic remodeling was most evident in selected macrophage/TAM states rather than uniformly across all myeloid cells (**Figures 4C, D**). This subset-specific interpretation is essential because broad statements about myeloid activation can mask functional differences between classical monocytes, macrophages, and TAM-like states.

The proportional increase in TAMs, together with enrichment of IL6–JAK–STAT3, TNFα–NFκB, hypoxia, angiogenesis, mTORC1-related programs, and checkpoint-associated genes, supports a model in which macrophage-lineage cells help establish an inflammatory, immunoregulatory, and metabolically active niche (**Figure 4E**). Prior work has implicated aberrant metabolic processes in the immunosuppressive MM microenvironment (1), and immune-checkpoint signatures have been observed in MM using single-cell and mass cytometry approaches (5). Our data connect these observations by placing macrophage/TAM states within a broader communication network that engages malignant plasma cells and dysfunctional T cells.

### Plasma-cell expansion is coupled to secretory stress and metabolic adaptation

The B/plasma-cell compartment exhibited the most prominent compositional alteration, with marked plasma-cell expansion and proportional reduction of memory B, naïve B, and PrePro B-cell populations (**Figure 5E**). This expansion was accompanied by activation of unfolded protein response, protein secretion, oxidative phosphorylation, MYC targets, and mTORC1-related programs (**Figure 5C**; **Supplementary Figure 4**). These findings are biologically consistent with the secretory burden of malignant plasma cells, which must sustain high immunoglobulin production while adapting to hypoxic, nutrient-constrained, and inflammatory marrow conditions.

This plasma-cell metabolic state is unlikely to be purely cell intrinsic. Increased oxidative phosphorylation, lipid metabolism, glycolytic/lactate metabolism, glutamine uptake, and mTORC1 signaling may enhance biosynthetic capacity and stress tolerance, while cytokine and checkpoint signals from surrounding stromal and immune compartments may further reinforce malignant plasma-cell survival. Prior studies have emphasized the role of metabolic adaptation in MM progression, drug resistance, glutamine addiction, LDHA-associated glycolytic programs, and bone marrow metabolic cross-talk (3, 4, 17, 18, 20–27). Our data extend this concept by linking plasma-cell metabolic stress to communication networks and immune suppression within the marrow ecosystem.

### Dendritic-cell and hematopoietic remodeling broaden the scope of MM-associated niche disruption

Dendritic-cell analysis showed reduction of CD1C DCs and a relative numerical increase in pDCs, accompanied by subset-dependent changes in interferon, inflammatory, IL6–JAK–STAT3, and mTORC1-related programs (**Figure 6**). Because conventional dendritic cells are central to antigen presentation and cytotoxic T-cell priming, depletion or functional alteration of these subsets may contribute to impaired antitumor immunity. The pDC-enriched pattern, although not statistically significant in all comparisons, is compatible with an immunoregulatory shift that may further dampen effective antigen presentation.

Beyond immune compartments, HSPC and blood-lineage populations also showed disease-associated trends. Erythroid, progenitor, neutrophil, and platelet-related compartments displayed altered inflammatory, metabolic, heme-metabolism, coagulation, and stress-response programs (**Figure 7**; **Supplementary Figure 6**). Several proportional changes did not reach statistical significance and should therefore be interpreted cautiously. Nevertheless, these data suggest that MM-associated remodeling extends beyond malignant plasma cells and immune subsets to include broader hematopoietic and blood-lineage perturbations. Prior evidence has shown that MM can deregulate CD34-positive hematopoietic stem and progenitor cells (28), supporting the concept that marrow hematopoiesis is shaped by tumor-derived and microenvironmental signals.

### Rewired communication networks define a TAM–plasma cell–T-cell regulatory circuit

Cell–cell communication analyses provide a systems-level explanation for how compartment-specific abnormalities may be coordinated. MM marrow showed disease-associated ligand–receptor networks involving malignant plasma cells, TAMs/macrophages, T/NK subsets, dendritic cells, platelets, and B-cell populations (**Figure 2**; **Figures 8A, B**). Communication-type analysis revealed a shift toward checkpoint-enriched and non-canonical interaction categories in MM, suggesting that disease-associated signaling is not restricted to soluble cytokines but includes immune-checkpoint, adhesion, integrin, extracellular-matrix, and other ligand–receptor programs (**Figure 8C**; **Supplementary Figures 7A, B**).

Several inferred axes are biologically plausible in the context of MM. TNFRSF17 and TNFRSF13B/C connect plasma-cell biology to B-cell maturation and survival programs; CD40LG-CD40 links activated T-cell states to B-lineage and antigen-presenting compartments; XCL1/XCL2-XCR1 connects cytotoxic or NK-like cells to dendritic-cell subsets; and PD-L1/LAG3-related programs connect myeloid/plasma-cell populations to T-cell dysfunction (29–32). Although CellChat inference alone does not prove causality, the convergence of communication analysis, marker expression, clinical association, and mIF validation supports the presence of a disease-associated immunoregulatory circuit.

This circuit is summarized in **Supplementary Figure 8B**. In this model, malignant plasma cells act as a metabolic and signaling hub, TAMs provide inflammatory and checkpoint-associated signals, and T cells acquire dysfunctional/exhaustion-associated states. IL6–JAK–STAT3 signaling, PD-L1, LAG3, TIGIT, HAVCR2, and ligand–receptor interactions such as TNFRSF17-associated signaling collectively define a self-reinforcing immunometabolic niche. This conceptual model does not imply that a single pathway fully explains MM biology; rather, it provides a framework for organizing the observed cellular, metabolic, communication, and validation data into a coherent disease mechanism.

### Clinical and functional validation support therapeutic relevance

The clinical analyses support the biological relevance of the inferred circuit. High plasma-cell-associated features and high CD274 expression were associated with inferior overall survival (**Figures 8D, E**). Serum IL-6 was significantly elevated in MM patients relative to normal controls in an independent clinical validation cohort (**Figure 8F**). Together, these observations connect three core elements of the proposed model: malignant plasma-cell expansion, checkpoint-mediated immune suppression, and IL-6-associated inflammatory signaling.

A major addition in the revised manuscript is the expanded biological and functional validation in **Figure 9**. Multiplex immunofluorescence confirmed the tissue-level presence of PD-L1-positive plasma cells, C1QA/C1QB/C1QC-positive TAMs with LAG3 expression, and CD8-positive/LAG3-positive cytotoxic T cells (**Figures 9A–C**). These spatially grounded observations support the coexistence of the cellular players inferred from single-cell and communication analyses.

The functional assays demonstrate that metabolic programs identified by single-cell analysis can be therapeutically perturbed. The choice of galloflavin and V-9302 was mechanistically anchored in the plasma-cell metabolic signature rather than empirically arbitrary. Galloflavin targets LDH-dependent glycolytic/lactate metabolism, corresponding to increased LDHA and related glycolytic genes in MM plasma cells; V-9302 targets ASCT2/SLC1A5-mediated glutamine transport, corresponding to increased SLC1A5, GLS, and glutamine-associated programs in the same compartment (14–20, 23, 24). Both agents reduced RPMI-8226 viability in a dose-dependent manner (**Figure 9F**). Combination testing showed synergistic anti-myeloma activity supported by positive Bliss synergy scores, CI values below 1, and observed inhibition exceeding Bliss-expected inhibition across tested combinations (**Figure 9G**). Time-course viability assays confirmed progressive suppression of cell viability, and Bax/Bcl-2 western blotting together with flow-cytometry apoptosis analysis supported induction of apoptotic programs after metabolic perturbation (**Figures 9H, I**). These findings suggest that malignant plasma cells may rely on parallel glycolytic/lactate and glutamine-dependent metabolic inputs, and that simultaneous disruption of these axes can expose a cooperative metabolic vulnerability.

These functional data also have therapeutic implications beyond the specific drug pair tested here. The transcriptional enrichment of lipid-metabolic genes, mTORC1-associated programs, and plasma-cell stress pathways suggests that glycolytic/lactate and glutamine-dependent metabolism may cooperate with other metabolic nodes, including fatty-acid synthesis and mTORC1 signaling, to sustain MM cell survival and immune suppression (33–35). This supports a broader rationale for testing combination strategies that pair metabolic inhibitors with immune-modulatory or plasma-cell-directed therapies in biomarker-defined MM contexts.

## Limitations and future directions

Several limitations should be acknowledged. First, the single-cell analysis was based on public datasets and was integrated with independent clinical, spatial, and functional validation rather than fully matched multi-omic sampling from the same patients. Second, CellChat and GSVA analyses provide inference of communication and pathway activity; they do not establish causality or direct metabolic flux. Third, serum IL-6 validation was performed in an independent cohort and should be interpreted as supportive clinical evidence rather than paired validation of the scRNA-seq dataset. Fourth, the *in vitro* functional assays were performed primarily in RPMI-8226 cells; additional MM cell lines, primary patient samples, ex vivo bone marrow cultures, and *in vivo* models will be needed to confirm broader applicability. Finally, although the integrated model highlights an IL6-centered TAM–plasma cell–T-cell circuit, individual ligand–receptor axes such as TNFRSF17-associated signaling, CD40LG–CD40, XCL1/XCL2–XCR1, and PD-L1/LAG3-related interactions require targeted perturbation to establish their relative mechanistic contribution.

Future studies should combine paired single-cell transcriptomics, spatial profiling, cytokine quantification, metabolic flux assays, stable-isotope tracing, and functional perturbation in patient-derived models. Such approaches would help determine whether IL6-JAK-STAT3 signaling, TAM-associated checkpoint programs, LDH-dependent glycolytic/lactate metabolism, or ASCT2/SLC1A5-mediated glutamine uptake can be used to stratify patients and guide rational metabolic-immune therapeutic combinations.

## Conclusion

In summary, our study provides an integrated view of MM bone marrow remodeling in which malignant plasma-cell expansion, TAM-associated inflammatory signaling, T-cell dysfunction, checkpoint-enriched communication, and metabolic stress converge into an IL6-centered immunometabolic circuit. By linking single-cell transcriptional states, ligand–receptor communication, clinical validation, spatial immunophenotyping, and functional metabolic perturbation, we identify a framework for understanding how immune suppression and metabolic reprogramming co-evolve in MM. These findings support further investigation of combined metabolic and immune modulation as a rational therapeutic strategy for MM.

## Data Availability

The original contributions presented in the study are included in the article/**Supplementary Material**. Further inquiries can be directed to the corresponding author.

## Supplementary material

Supplementary Figure 1 Full cell-cell interaction-strength matrices in MM and HV bone marrow. **(A)** Interaction-strength matrix for MM. **(B)** Interaction-strength matrix for HV. Heatmaps show inferred CellChat interaction strength between all annotated sender and receiver cell populations. Rows and columns correspond to annotated cell types, and each matrix entry represents the predicted interaction strength for the corresponding cell-type pair. Higher values indicate stronger inferred communication; NA indicates unavailable or non-estimated interactions. This supplementary figure provides the complete interaction-strength background supporting the summarized communication networks in **Figures 2** and **9**.

Supplementary Figure 2 Full Hallmark GSVA activity landscape across T/NK-cell subsets. Heatmap of GSVA scores for the complete MSigDB Hallmark pathway collection across T/NK subtype-cohort combinations. Columns correspond to T/NK subsets stratified by HV or MM, and rows correspond to Hallmark pathways. Column annotations indicate cell type and cohort. Color represents scaled pathway activity. This figure provides the full pathway context for the selected immune, inflammatory, metabolic, and exhaustion-associated programs emphasized in **Figure 3**.

Supplementary Figure 3 Full Hallmark GSVA activity landscape across monocyte/macrophage subsets. Heatmap of GSVA scores for all MSigDB Hallmark pathways across CD14 monocytes, macrophages, and TAMs from HV and MM. Columns represent cell subtype-cohort combinations, and rows represent Hallmark pathways. Color indicates scaled pathway activity. This figure supports the selected pathway modules shown in **Figure 4**, including inflammatory signaling, IL6-JAK-STAT3 activation, complement remodeling, hypoxia, angiogenesis, and metabolic stress-response programs.

Supplementary Figure 4 Full Hallmark GSVA activity landscape across B-cell and plasma-cell subsets. Heatmap of GSVA scores for all MSigDB Hallmark pathways across memory B cells, naive B cells, plasma cells, and PrePro B cells in HV and MM. Columns represent cell subtype-cohort combinations, and rows represent Hallmark pathways. Color indicates scaled pathway activity. This supplementary view provides the full background for interpreting plasma-cell-associated UPR, protein secretion, oxidative phosphorylation, MYC, mTORC1, and inflammatory pathway activity in **Figure 5**.

Supplementary Figure 5 Full Hallmark GSVA activity landscape across dendritic-cell subsets. Heatmap of GSVA scores for all MSigDB Hallmark pathways across CD1C DCs, CD14 DCs, CLEC9A DCs, and pDCs in HV and MM. Columns represent cell subtype-cohort combinations, and rows represent Hallmark pathways. Color indicates scaled pathway activity. This complete pathway matrix supports the selected dendritic-cell programs shown in **Figure 6**, including interferon responses, inflammatory signaling, complement, apoptosis, and mTORC1-associated remodeling.

Supplementary Figure 6 Full Hallmark GSVA activity landscape across HSPC and blood-lineage subsets. Heatmap of GSVA scores for all MSigDB Hallmark pathways across erythroblasts, erythrocytes, erythroid progenitors, HSPCs, immature neutrophils, and platelets in HV and MM. Columns represent cell subtype-cohort combinations, and rows represent Hallmark pathways. Color indicates scaled pathway activity. This figure provides the full pathway context for hematopoietic stress, erythroid and platelet biology, inflammatory signaling, heme metabolism, coagulation, proliferation, and metabolic remodeling summarized in **Figure 7**.

Supplementary Figure 7 Cell-cell communication networks stratified by communication type. **(A)** Chord diagram of inferred cell-cell communication in HV bone marrow stratified by communication category. Cell types are arranged around the circle, and connecting ribbons represent predicted ligand-receptor interactions. Ribbon color denotes communication type, including checkpoint, cytokine, and other categories. This panel provides a categorical view of baseline healthy marrow communication. **(B)** Chord diagram of inferred cell-cell communication in MM bone marrow stratified by the same communication categories. Comparison with panel A illustrates disease-associated redistribution of checkpoint, cytokine, and other interaction classes, highlighting the altered communication architecture that underlies the MM immunometabolic niche.

Supplementary Figure 8 Integrated immunometabolic remodeling and conceptual model of the IL6-centered communication circuit in MM. **(A)** Integrated Hallmark pathway remodeling across major bone marrow cell compartments. Dot plot summarizing representative Hallmark pathway activities across T/NK cells, monocyte/macrophage/TAM populations, B/plasma-cell compartments, dendritic cells, and HSPC/blood-lineage populations. Dot color represents the relative pathway activity difference between MM and HV (Delta GSVA score, MM minus HV), whereas dot size reflects statistical significance (-log10[FDR]). Recurrently enriched pathways include inflammatory signaling, TNFalpha-NFkappaB, IL6-JAK-STAT3, oxidative phosphorylation, mTORC1 signaling, protein secretion/UPR, hypoxia, and angiogenesis, indicating coordinated immunometabolic remodeling across multiple marrow compartments. **(B)** Conceptual model of the proposed immunometabolic circuit in MM. Malignant plasma cells interact with macrophages/TAMs and dysfunctional T cells through ligand-receptor signaling and metabolite exchange. Plasma-cell-associated molecules, including TNFRSF17 and SDC1, together with macrophage/TAM-derived inflammatory mediators such as IL-6, contribute to activation of immune-checkpoint programs, including PD-L1, LAG3, TIGIT, and HAVCR2. These interactions establish a self-reinforcing metabolic-immune suppression circuit that promotes immune evasion, sustains tumor-supportive communication networks, and creates potential therapeutic vulnerabilities for metabolic intervention.
